# Heat-Induced Structural Changes in Lactoferrin for Enhanced Mucoadhesion

**DOI:** 10.1021/acsabm.5c01534

**Published:** 2025-10-18

**Authors:** Bianca Hazt, Daniel J. Read, Oliver G. Harlen, Wilson C. K. Poon, Adam O’Connell, Simon D. Connell, Anwesha Sarkar

**Affiliations:** † Food Colloids and Bioprocessing Group, School of Food Science and Nutrition, 4468 University of Leeds, Leeds LS2 9JT, U.K.; ‡ School of Mathematics, 4468 University of Leeds, Leeds LS2 9JT, U.K.; § School of Physics and Astronomy, 3124 University of Edinburgh, Edinburgh EH9 3FD, U.K.; ∥ Polymer Science Platform, 544023 Reckitt Benckiser Healthcare (U.K.) Ltd., Hull HU8 7DS, U.K.; ⊥ Molecular and Nanoscale Physics Group, School of Physics and Astronomy, 4468 University of Leeds, Leeds LS2 9JT, U.K.

**Keywords:** mucin, protein aggregation, denaturation, QCM-D, hydrophobic interaction

## Abstract

The development of biocompatible and safe mucoadhesive materials is critical for improving therapeutic strategies, where cationic proteins such as lactoferrin are emerging as promising alternatives to synthetic polymers. Here, we demonstrate how thermal denaturation of lactoferrin can be used as a viable strategy to enhance mucoadhesion. We identify and study in detail the structural changes in lactoferrin upon thermal denaturation using light scattering, circular dichroism spectroscopy, gel-electrophoresis, and atomic force microscopy. Lactoferrin-mucin binding was evaluated using rheology, confocal microscopy, and quartz crystal microbalance with dissipation monitoring. We find that lactoferrin binds to mucin at its native state, heat-treatment at 95 °C enhances its affinity for mucin, and that the adhesion mechanism relies on hydrophobic interactions with no obvious contributions of disulfide bonds. Lactoferrin and its resulting complexes with mucin present high surface activity, which induces an artificial shear-thinning rheological response. While electrostatic interactions have been considered the dominant mucoadhesive mechanism of native lactoferrin up to now, our findings highlight the role of hydrophobic interactions, providing a design route to alter the structural state of the protein to inspire the development of future natural protein-based mucoadhesive systems.

## Graphical abstract

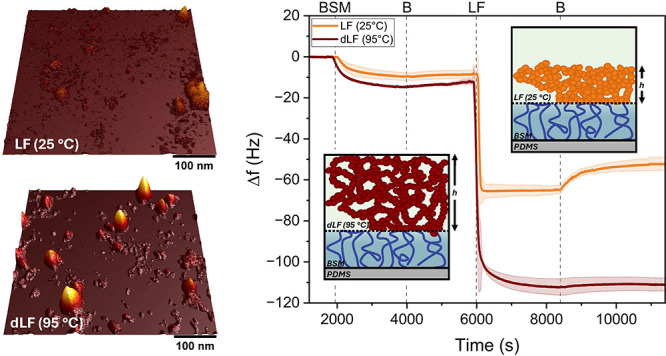

## 1 Introduction

Mucoadhesion, the phenomenon by which materials adhere to soft mucosal surfaces, plays a critical role in a wide range of applications spanning pharmaceutical, biomedical, and food sciences.^1^ Given the ubiquitous presence of mucus throughout the body, understanding and engineering strong mucoadhesive interactions is fundamental for advancing targeted drug delivery systems and developing muco-protective coatings. Mucin, the primary structural component of mucus apart from water, is a highly glycosylated protein rich in functional groups that facilitate diverse intermolecular interactions,^2^ with an isoelectric point (IEP) between 2 and 3.^3^ The mucoadhesive properties of a range of materials have been explored in literature, particularly for synthetic polymers, as well as some chemically modified proteins such as gelatin modified with unsaturated anhydrides^4^ or bovine serum albumin modified with *N*-Acetylcysteine.^5^ A synthetic amphoteric molecule obtained with polyethylene imine-succinic or phthalic anhydrides has also been investigated and exhibited strong mucoadhesive properties for pH < IEP.^6^ In contrast, naturally occurring amphoterics such as the protein lactoferrin have received comparatively less attention,^7^ despite their potential as a biocompatible and multifunctional mucoadhesive agent.

Lactoferrin (LF), an iron-binding protein with host defense properties^8^ is found in the secretions of mammals such as tears, saliva, and milk. Bovine LF has five glycosylation sites,^9^ and between 10 and 30% of the total protein contains bound iron (holo-LF), which gives it a characteristic red color.^10^ Its molecular weight is in the range of 80 kDa,^11^ its native isoelectric point (IEP) is around pH 8 ^12^ and the reported denaturation temperatures range from 61 to 82 °C, depending on the ferric saturation.^10^ Peak denaturation temperatures reported in literature vary between 60 and 61 °C for apo-LF (the iron-free form), and 89–91 °C for holo-LF. Due to the presence of both iron-saturated and iron-depleted lobes, native LF presents both denaturation temperature ranges.^10^

LF has gained increasing interest in recent years for its interactions with mucins and promise as mucoadhesive material. Previous studies suggest that native LF-mucin binding is primarily governed by electrostatic interactions, particularly Coulombic forces between positively charged regions of LF and the negatively charged domains of mucin^13^ at physiologically relevant pH. However, lactoferrin also contains various reactive sites within its molecular structure, providing opportunities for conjugation with functional groups in mucin which have remained principally unexplored. Of particular importance, the extent to which these interactions are modulated by structural modifications in LF remains poorly understood. Thermal treatment is a simple, physical route known to induce conformational changes in proteins, altering their surface charge distribution, hydrophobicity, and aggregation state. A well-documented consequence of protein denaturation is the exposure of buried hydrophobic residues, which can significantly impact interfacial interactions.^14^ Upon heat-treatment, LF undergoes structural unfolding toward a more flexible conformation, with hydrophobic residues exposed and the formation of aggregates.^15^ While knowledge of the role of heat treatment on iron-binding properties of lactoferrin is fairly complete, understanding of its effect on mucin interactions is at a nascent stage. To harness the full potential of LF as a mucoadhesive material, it is imperative to understand how heat-induced alterations in LF structure may change its mucoadhesive properties.

In this study, we investigate the impact of thermal treatment on LF-mucin interactions using a combination of quartz crystal microbalance with dissipation monitoring (QCM-D), confocal laser scanning microscopy (CLSM), and shear rheometry. QCM-D provides real-time insights into adsorption kinetics, viscoelastic properties, and hydrated mass of the LF-mucin complex, while rheological measurements assess the bulk mechanical behavior of the resulting LF-mucin networks. To elucidate the structural changes in LF following heat treatment, we employ dynamic light scattering (DLS), circular dichroism (CD), sodium dodecyl sulfate-polyacrylamide gel electrophoresis (SDS-PAGE), asymmetric flow field-flow fractionation (AF4), and atomic force microscopy (AFM).

By systematically evaluating the effects of thermal processing on LF structure and its ability to interact with mucins, this work provides insights into the molecular mechanisms underpinning protein-mucin adhesion. These findings hold broad implications for the rational design of protein-based mucoadhesive formulations in biomedical applications, where precise control over bioadhesion is essential for optimizing functional performance.

## 2 Materials and Methods

### 2.1 Materials

Bovine LF was purchased from Ingredia Dairy Experts (Arras, France) (Proferrin, batch number U21008, protein content >93%, LF content of protein content >95%) and used without further purification. Mucin from bovine submaxillary glands (BSM) (Type I–S, lot number SLCJ8335) was acquired from Sigma-Aldrich, Dorset, UK and purified before use. HEPES (4-(2-hydroxyethyl)-1-piperazineethanesulfonic acid) salt (A1069, Lot number 1D011080) for preparation of buffer solutions was purchased from ITW Reagents, Monza, Italy. Sodium hydroxide (NaOH) was used for pH adjustment. SDS-PAGE experiments were conducted following the NuPAGE Electrophoresis System protocols and reagents, as detailed further below. Ellman’s Reagent (Sigma-Aldrich, ≥ 98%) was used to quantify thiol groups. Ultrapure water (18 M Ω cm at 25 °C, purified using Milli-Q apparatus, Millipore Corp., Bedford, MA, USA) was used to prepare the buffer solutions or to disperse LF, as described in the methods section.

### 2.2 Methods

#### Sample Preparation

HEPES buffer solutions were prepared at 10 mM and pH 7.0, and are referred to as “HEPES buffer”, “HEPES” or simply “buffer” hereafter. LF samples were prepared by slowly dispersing the protein powder in HEPES buffer solutions, under magnetic stirring, for 2 h to ensure complete dispersion. Fresh solutions were prepared each day. Heat-treated LF samples were obtained by heating LF dispersions in a water bath (Sub Aqua Pro, Grant Instruments Ltd., Cambridge, UK) with the temperature set to the appropriate temperature for 30 min. These temperatures were chosen as they are associated with LF’s denaturation^10^: 65 °C is above the first denaturation temperature (of 60–61 °C), 95 °C is beyond the last denaturation peak (89–90 °C), and 80 °C is in between both. The resulting samples are LF (25 °C) for the native one, dLF (65 °C) for the sample heat-treated at 65 °C, dLF (80 °C) for the sample heat-treated at 80 °C, and dLF (95 °C) for the sample heat-treated at 95 °C (where dLF denotes denatured LF).

BSM was purified prior to use to remove other protein impurities^16^; the protocol consisted of dialysing BSM against ultrapure water for 10 days, with at least 3 water changes per day, using 100 kDa molecular weight cutoff membranes (Spectra/Por Float-A-Lyzer G2). The dialyzed BSM was lyophilized (FreeZone 2.5, Labconco Corp., Kansas City, MO, USA) and stored at −18 °C until further analyses.

#### Circular Dichroism

Circular Dichroism (CD) Spectroscopy was used to investigate possible changes in the secondary and tertiary structures of LF after thermal treatment, using a Chirascan spectrometer (Applied Photophysics, Leatherhead, UK), at 25 °C. The blank consisted of a spectrum acquired only for HEPES buffer or ultrapure water in the cuvette, which was subtracted from each protein sample spectra. Each measurement was performed twice in triplicate (*n* = 3 × 2) and the mean of three CD spectra was considered for each sample. Samples were prepared by dispersing LF in HEPES buffer or ultrapure water at a concentration of 0.2 wt % (equivalent to a volume fraction, ϕ = 0.004, or molar concentration of ∼25 μmol L^–1^). Heat-treated samples were prepared by placing the samples on a water bath, at 65, 80, or 95 °C for 30 min, as described above. Sample raw ellipticity (mdeg) data was plotted against wavelength (nm) as all samples were prepared at the same concentration, and any increase in molecular weight for aggregated samples is followed by an increase in the number of residues per aggregate.

For the secondary structure determination, each sample was loaded into the same 0.1 cm path length quartz cuvette. The CD spectra were recorded using 1 nm intervals, in the far-UV region, from 180 to 260 nm. For the tertiary structure, the near-UV region (250 to 340 nm) was investigated, using 1 nm steps and a 1.0 cm path length quartz cuvette.

#### Determination of Hydrodynamic Diameter (*d*_H_)

The hydrodynamic diameter (*d*_H_) was determined using dynamic light scattering (DLS) on a Zetasizer Ultra (Malvern Instruments, Worcestershire, UK), equipped with a 10 mW He–Ne 632.8 nm laser, at 173° (back scattering). The samples were prepared at a total protein concentration of 0.01 wt % (volume fraction ϕ = 0.0002, or molar concentration of ∼1.25 μmol L^–1^) in HEPES buffer at 25 °C and filtered by hydrophilic PTFE filters with 0.22 μm pore size (HPF Millex) before being transferred to a synthetic quartz glass cuvette (10 mm of path length).

Measurements were performed by varying the temperature from 25 to 50 °C and from 65 to 95 °C in 5 °C steps, and from 50 to 65 °C in 1 °C steps, as this is the temperature range where protein denaturation was identified. The instrument required around 2 min to reach each target temperature, after which the sample was held for an additional 5 min to allow for temperature equilibration prior to measurement. For the in situ heating of 2 mL samples of dLF (65, 80, or 95 °C), no differences in particle size distribution were observed when the equilibration time was extended to 30 or 60 min. This indicates that the protein self-assembly reached a steady state within 5 min under these conditions. Moreover, external heat-treatment of 60 mL samples in a water bath for 30 min followed by DLS measurements led to similar results of particle size distribution, suggesting that the scale and method of heating did not significantly affect the final self-assembled state as measured by DLS. When the laser illuminates the particles in solution, a speckle pattern is generated by the interference of scattered light. Based on the time-dependent fluctuations of this speckle pattern, an auto correlator generates an intensity correlation function. The ZS Explorer software employs a cumulant analysis method by fitting a single exponential to this correlation function, obtaining the translational diffusion coefficient of the colloidal material, allowing the calculation of the hydrodynamic, *d*_*h*_, based on the Stokes–Einstein eq 1,

$$d_{h}=\frac{k_{B}T}{3\pi \eta D}$$ 1

where, *D* is the diffusion coefficient, *k*_*B*_ is the Boltzmann constant, *T* is the temperature and η is the bulk viscosity of the solution. The refractive index of the solvent was set to 1.33, the default value provided by the Zetasizer software. Protein absorbance was 0.001 (at 0.01 wt % protein concentration), which was confirmed using a UV–vis spectrophotometer at 633 nm. Two replicates, each measured in triplicate were used, and each value is presented here as the mean value and standard deviation of six measurements (*n* = 3 × 2), represented according to the calculated volume distribution of hydrodynamic diameters.

#### Determination of ζ-Potential

The ζ-potential values of LF or dLF samples were measured using standard folded capillary electrophoresis cells (DTS1070), on a Zetasizer Ultra (Malvern Instruments Ltd., Worcestershire, UK). LF dispersions were measured at a protein concentration of 0.001 wt %. Samples were filtered using a hydrophilic PTFE filter with 0.22 μm pore size (HPF Millex) prior to the measurements. The software ZS Explorer automatically converts the electrophoretic mobility measurements (*U*_*E*_) into ζ-Potential values by considering the Smoluchowski approximation that *f*(κα) = 1.0 when using the Henry’s eq 2,

$$U_{E}=\frac{2\varepsilon \zeta f\left(\kappa \alpha \right)}{3\eta }$$ 2

where, ε is the dielectric constant, ζ is the ζ-potential value, κ is the inverse of the Debye screening length, and α is the particle radius. Each sample was measured in triplicate, and the reported values represent the mean value accompanied by the standard deviation of six readings (*n* = 3 × 2).

#### Atomic Force Microscopy (AFM)

Lactoferrin dispersions (heated at 65, 80, or 95 °C and unheated) at 1 wt % were diluted 2000x using serial dilutions using HEPES buffer at pH 7.0. 50 μL of each sample were deposited onto freshly cleaved mica discs and incubated for 10 min, allowing sample adsorption by diffusion onto the mica. Ultrapure water was used to rinse any remaining salt from the samples (3 mL), followed by a N_2_ stream.^17^ Sample adsorption is promoted by electrostatic interactions, as LF is positively charged at pH 7.0, whereas freshly cleaved muscovite mica presents negatively charged silicate groups.^18^ Samples were scanned using a Multimode 8 AFM equipped with a Nanoscope V controller (Bruker Nano Surfaces, Santa Barbara, CA) and TESPA-V2 probes (Bruker). Images were acquired using tapping mode in air, with a resonant frequency of 320 kHz, an amplitude set point of ∼500 mV, and scan rates of 1–4 Hz. Multiple scans of each sample were obtained; at 5, 2 μm or 500 nm sizes. AFM images were analyzed using Nanoscope Analysis software (Bruker, version 3.0) and subjected to second order flattening with thresholding, and particle analysis was carried out using NanoLocz,^19^ by detecting the full width half maxima of 5–6 images from different locations (3 images in the case of dLF (80 °C)). Particle analysis statistics were aggregated from a combination of 3 μm, 1 μm and 500 nm images, where individual LF could only be discriminated at 1 μm or 500 nm scan size.

#### Determination of *R*_*g*_ and *M*_*w*_ (AF4-MALS)

Molar mass (*M*_*w*_) and radius of gyration (*R*_*g*_) values for the purified BSM were determined via asymmetrical flow field-flow fractionation (AF4), using an AF200 multiflow system (Postnova Analytics, Malvern, UK). The system was connected to RI (PN3150), MALS (PN3621), and UV (SPD-20A) detectors. RI and MALS were set at 532 nm, and UV at 220 and 280 nm. Separation was conducted using a membrane made of regenerated cellulose with a 10 kDa cutoff and a spacer of 350 μm. RI, MALS and UV data were collected and analyzed with the NovaFFF software version 2.0.9.9 (Postnova Analytics, Worcestershire, UK). *M*_*w*_ and *R*_*g*_ were determined in the low scattering angle limit using the Zimm plot.

Purified BSM was dispersed at 5 g L^–1^ in the carrier liquid; 13 μL in total were injected. The carrier liquid was NaCl at 0.01 mol L^–1^ which had been previously filtered through a 0.1 μm hydrophilic filter. The sample was filtered through a 1 μm hydrophilic filter prior to injection. The d*n*/d*c* value used was 0.144.^20^ Two samples were injected in triplicate (*n* = 3 × 2); *M*_*w*_ and *R*_*g*_ values were calculated considering all six replicate runs. The elution protocol consisted of three elution stages and was previously described in detail by Collado-Gonzalez and coauthors.^20^ Briefly, a constant detector flow rate of 0.5 mL/min was used, with 2.5 mL/min as the flow rate of the focus and a focusing time of 3 min. At the first elution stage, a constant 2.5 mL/min of cross-flow for 0.2 min was used; followed by the reduction of the cross-flow to 0.2 mL/min in a power decay mode with an exponent of 0.25 in 20 min; to 0.12 mL/min in 5 min with an exponent of 0.8; and to 0.09 mL/min in 5 min with an exponent of 0.8. A blank signal was obtained for pure carrier liquid and subtracted from the recorded signals from mucins for all detectors.

#### Determination of *M*_*w*_ (SDS-PAGE)

Sodium dodecyl sulfate-polyacrylamide gel electrophoresis (SDS-PAGE) was used to characterize the molecular weight of BSM or LF samples with or without heat-treatment (65, 80, and 95 °C for 30 min). 75 μL of each sample (at 0.2 wt %) were mixed with 25 μL of the Nu-PAGE LSD sample buffer and incubated for 10 min at 70 °C, which allow for migration based on the molecular weight of the protein instead of its charge or shape. The SDS-PAGE was carried out by loading 10 μL of protein marker (Invitrogen Novex Sharp Pre-Stained Protein Standard) or 10 μL of each sample in each well, using an Invitrogen Mini Gel Tank system as the electrophoretic unit connected to a PowerEase 90W power supply. NuPAGE MES SDS was used as a running buffer, and NU-PAGE 4–12% Bis–Tris gel was used to separate the protein fractions. The running process took 36 min, at a constant voltage of 200 V. After the run, each gel was stained overnight using the SimplyBlue Coomassie G-250 safe stain), washed with ultrapure water and imaged on a ChemiDoc XRS+ Imager connected to the software Image Lab 5.0 (Bio-Rad Laboratories, Inc., USA), using the Cubic-Spline regression method to determine the *M*_*w*_ values. Separate gels were run without DTT (noncovalent reducing conditions) and with DTT (covalent reducing conditions).

#### Determination of Thiol Groups

Free thiol groups in BSM or LF with or without heat-treatment (65, 80, 95 °C, 30 min) were determined using the Ellman’s reagent (5.5′-dithiobis­(2-nitrobenzoic acid) (DTNB), which reacts with free sulfhydryl groups and forms a yellowish product, quantifiable at 412 nm. For the assay, 250 μL of each sample at 0.1 or 1 wt % were mixed with 2.5 mL of phosphate buffer at pH 8.0 with 1 mM of EDTA, and with 50 μL of DTNB (at 0.4 wt %). The samples were incubated for 2 h at 25 °C protected from light. The absorbance was then measured at 412 nm using a Spark multimode microplate reader (Tecan, Männedorf, Switzerland). The calibration curve was constructed following the sample procedure, using homocysteine as a standard with concentrations from 0.1 to 1 mmol L^–1^. Quantification was performed in triplicate for three samples (*n* = 3 × 3) and results are reported as mean ± standard deviation.

#### Determination of Surface Hydrophobicity Index

LF (25 °C) or dLF (65, 80, or 95 °C) samples were diluted to concentrations ranging between 0.005 and 0.01 wt % in 10 mmol L^–1^ HEPES at pH 7. A total volume of 4 mL was prepared in each case and mixed with 10 μL of 8-Anilinonaphthalene-1-sulfonic acid (ANS) at 8 mmol L^–1^. Mixtures were incubated at 25 °C for 15 min and the fluorescence emission was recorded using a FluoroMax-4 Spectrofluorometer (Horiba, Northampton, UK), using an excitation wavelength of 390 nm and recording the emission from 400 to 600 nm. The maximum absorbance was recorded at 492 nm. Each set of data was fitted to a linear function where the slope corresponds to the surface hydrophobicity index (*H*_0_). Measurements were performed in triplicate for three samples (*n* = 3 × 3) and results are reported as mean ± standard deviation for all nine readings.

#### Quartz Crystal Microbalance with Dissipation Monitoring (QCM-D)

##### Sensor Cleaning and Surface Preparation

QSense SiO_2_ sensors (QSX 303, Biolin Scientific, Sweden) were coated with PDMS following the method described previously.^21^ Briefly, sensor substrates were first cleaned via UV/ozone treatment for 15 min, followed by immersion in 95% sulfuric acid for 1 h. Subsequently, substrates were sonicated twice in ultrapure water for 10 min and dried under a nitrogen stream. Sensors were then immersed in an RCA solution (5:1:1 v/v ultrapure water: ammonia: hydrogen peroxide) at 80 °C for 10 min, followed by three sonication cycles in ultrapure water (10 min each). Cleaned substrates were coated with 150 μL of a 10 wt % solution of PDMS (Sylgard 184) in toluene using spin coating at 5000 rpm for 60 s (acceleration: 2500 rpm s^–^^1^). Coated sensors were left for overnight curing and toluene evaporation in a vacuum oven at 80 °C overnight. Prior to the QCM-D measurement, PDMS-coated sensors were immersed in toluene (30 s), isopropanol (30 s) and ultrapure water (5 min), before drying with nitrogen.

##### QCM-D Measurements

Before dispersing LF and BSM samples on HEPES buffer, the buffer solutions were degassed using an ultrasonic bath (XUB18, Grant Instruments, Cambridge, UK) for 10 min, to avoid the interference of air bubbles in the QCM-D experiment. LF or dLF at 0.01 wt % and BSM at 0.1 wt % were prepared in HEPES buffer. During the QCM-D measurement using a Q-Sense Analyzer (Biolin Scientific, Sweden), the solutions were injected using a peristaltic IPC High-precision multichannel pump (VWR, Leicestershire, UK) with a flow rate of 100 μL/min, at 25 °C. After a stable baseline was observed (around 30 min after t_0_), the BSM solution was injected, followed by a rinsing step with HEPES buffer to ensure that loosely attached materials were washed away. To avoid air bubbles entering the sensor chambers, the flow was stopped when changing solutions. LF samples were subsequently injected, followed by HEPES buffer rinsing as a last step. The real-time changes in dissipation (Δ*D*) and frequency (Δ*f*) were recorded as a function of time with the different solutions being injected. The results are shown as the mean ± standard deviation values of at least 6 measurements, using two different samples for each curve with either LF (25 °C), or heat-treated LF (65, 80, or 95 °C). Biolin’s Dfind software was used to fit the experimental data to the Voinova model^22^ and obtain the final hydrated mass on each sensor. According to the Voinova model, the change in frequency (Δ*f*) and dissipation (Δ*D*) are given by,

$$\Delta f\approx -\frac{1}{2\pi \rho_{Q}h_{Q}}\left\{\frac{\eta }{\delta }+\left[h_{\mathrm{layer}}\rho_{\mathrm{layer}}\omega -2h_{\mathrm{layer}}{\left(\frac{\eta }{\delta }\right)}^{2}\frac{G_{\mathrm{layer}}^{\prime \prime }\omega^{2}}{{G_{\mathrm{layer}}^{\prime }}^{2}+{G_{\mathrm{layer}}^{\prime \prime }}^{2}\omega^{2}}\right]\right\}$$ 3

$$\Delta D\approx -\frac{1}{2\pi f\rho_{Q}h_{Q}}\left\{\frac{\eta }{\delta }+\left[2h_{\mathrm{layer}}{\left(\frac{\eta }{\delta }\right)}^{2}\frac{G_{\mathrm{layer}}^{\prime }\omega }{{G_{\mathrm{layer}}^{\prime }}^{2}+{G_{\mathrm{layer}}^{\prime \prime }}^{2}\omega^{2}}\right]\right\}$$ 4

In (3) and (4) η is the bulk fluid viscosity and δ the shear wave penetration depth; *h*_layer_ is the thickness and ρ_layer_ is the density of the layer; ω is the angular frequency and *G*′ and *G*″ are respectively the storage and loss moduli for the layer.

##### Contact Angle Measurements

Before and after film formation on the QCM-D SiO_2_ sensors, static water-contact angle measurements were performed using a drop-shape analysis device (OCA, Dataphysics, UK). After the QCM-D experiment, each sensor was left for at least 12 h for liquid evaporation at room temperature (22 °C) before the contact angle measurement. Approximately 10 μL of ultrapure water was dispensed through a needle, and the mean angle was determined by the right and left contact angles of the water droplet imaged by the camera. For the contact angle determination, three readings were obtained for each sensor.

##### Confocal Laser Scanning Microscopy (CLSM)

Samples (LF or dLF and BSM) were dispersed at the desired concentrations in HEPES buffer with different staining agents. Fast green FCF was used for LF and calcofluor white for BSM, both at 200 ppm. Confocal laser scanning microscopy images were acquired using an inverted Zeiss LSM880 microscope (Oberkochen, Germany) equipped with 40× or 63× oil-immersed objective lens, with frame sizes of 1024 × 1024 pixels. Calcofluor white and fast green were detected using excitation and emission wavelengths of 360 nm/450 and 633 nm/675 nm, respectively. 250 μL of each mixture were placed at glass-bottom μ-slides with 8 wells covered with a lid, and the images were acquired within 2 h after sample preparation. A scale bar was included in the images using Fiji (ImageJ) software.

##### Viscosity Measurements

To measure the viscosity of aqueous solutions containing LF and LF-BSM as well as dLF-BSM complexes, an Anton Paar MCR 302 (Anton Paar Ltd., Hertfordshire, UK) stress-controlled rheometer was used with a cylindrical double-gap (DG) geometry (DG27/T200/SS) which allows the measurement of low viscosities such as that of water because of the high contact area between the sample and the geometry. The double gap geometry has an inner cup diameter of 23.039 mm, outer cup diameter of 29.282 mm, a bob inner diameter of 24.997 mm and outer diameter of 27.007 mm. A cone-and-plate geometry (CP50-1, 49.955 mm in diameter, 0.996° cone angle, 1 mm gap) and a bicone geometry (BiC68-5) were also used to check for the presence of an interfacial viscoelastic film contributing to torque readings.

LF samples were prepared by dispersing LF in HEPES buffer at 1.0 wt % (ϕ = 0.02). Heat-treated dLF samples were prepared by heating this 1.0 wt % LF sample using a water bath (Grant, SUB Aqua Pro) set to 65 °C, 80 or 95 °C for 30 min. BSM samples were prepared by dispersing BSM at 1.0 wt % in HEPES solution. LF/BSM complexes were prepared by mixing 1.0 wt % LF or dLF and BSM stock solutions, at equal proportions.

Each sample was loaded into the selected geometry using a plastic pipet and left for 5 min for thermal equilibration at 25 °C, set using a temperature control system (P-PTD200+H-PTD200). All samples were presheared at 500 s^–1^ for 60 s and then sheared at 0.1 s^–1^ for 360 s to ensure shear history similarity between replicates. We estimated that different shear histories (7–40 s^–1^) are imposed in the sample depending on the pipetting time, when using disposable Pasteur pipettes. This is relevant because as discussed in the results section, the shear history in aqueous samples containing LF or dLF lead to differences in the measured shear stress responses. The measurements were performed at shear-rates from 1 to 100 s^–1^. At higher shear rates (>150 s^–1^) flow instabilities were detected. The time-out limits used were 300 s from 1 to 10 s^–1^ and 30 s from 10 to 100 s^–1^. As discussed in the results section, this was sufficient to avoid transient effects in the measurement of the steady-state viscosity. Viscosity data are reported as the means of two readings for three independent samples.

##### Statistical Analysis

The reported values represent the mean ± standard deviation of at least three independent measurements on duplicate samples (*n* = 3 × 2), unless otherwise specified.

## 3 Results and Discussion

### Thermal Treatment Induces Conformational Changes in LF

Generally, protein aggregation in food systems can occur resulting in many structures such as amyloid-like fibrils, fractal or amorphous aggregates,^23^ depending on the protein and environmental conditions. In the present study, however, we use the term ‘aggregation’ specifically to describe the self-association of lactoferrin induced by temperature and focus on its interplay with mucin. Therefore, before exploring interactions with mucin, we first present experimental evidence demonstrating the effect of heat-treatment on the structure and behavior of LF.

For globular proteins such as LF, the typical conformational free energy variation (Δ*G*_*c*_) from the native to the denatured state involves 5 to 15 kcal mol^–1^, as determined in protein denaturation thermodynamics studies,^24^ which is equivalent to a few hydrogen bonds being broken,^24^ and leads to the loss of protein’s native conformation. In addition to structural unfolding, this loss may also be accompanied by aggregation.^14^ This discussion section will begin by addressing the impact of heat treatment on the structural properties of lactoferrin (LF), followed by alterations to its size, aggregation pattern, and surface characteristics such as net charge and the exposure of functional groups.

Here, the alterations in the structural elements after heating the LF were followed by CD spectroscopy, based on the absorption of circularly polarized UV light. Figure 1a shows the far-UV region which typically detects secondary structure. An inset shows the characteristic signals for secondary structure elements such as α-helices which has a double minima around 209 and 221 nm and β-sheets a single minimum near 216 nm.^25^ In the native state, no significant differences were observed when comparing the secondary structure of LF in HEPES buffer (pH 7.0) or in ultrapure water (SI, Figure S1a). The spectrum for the pristine LF resembles the native aspect of this protein in terms of secondary structure described in the literature,^10,26,27^ with two negative peaks corresponding to α-helices at 209 and 221 nm for the unheated LF. Following heat treatment, dLF shows a reduction in the signal magnitude of these peaks indicating the loss of α-helical structures, which is in agreement with previous reports.^15^ Quantitative analysis using BeStSel^28^ indicated α-helix content decreased from 18 to 12%, corresponding to a relative 33% decrease when comparing LF (25 °C) with dLF (95 °C) (Figure S2). The loss of β-sheets, when in HEPES buffer, is also noted from the change in signal at 216 nm (Figure 1a). Secondary structure analysis with the BeStSel method revealed distinct alterations in β-sheet composition, with only a minor reduction in antiparallel β-sheets but a pronounced decrease (>50%) in parallel β-sheets (Figure S2). This effect on the loss of β-sheets was less pronounced for the sample in water, indicating the buffer salt contribution in destabilizing the elements of secondary structure and inducing protein unfolding (SI, Figure S1b). In addition to ionic screening effects, the presence of physiological salts has been associated with iron release from lactoferrin,^29^ which significantly alters LF’s structural conformation.^30^ It is also important to highlight that complete denaturation, i.e., near-complete loss of secondary structure elements (as reported by Barrios et al. in the presence of sodium citrate^31^), was not achieved under our conditions. Nevertheless, heat treatment induces marked alterations in tertiary structure and aggregation behavior, as demonstrated by complementary analyses (near- UV CD, DLS, and AFM; see below).

**Figure 1:**
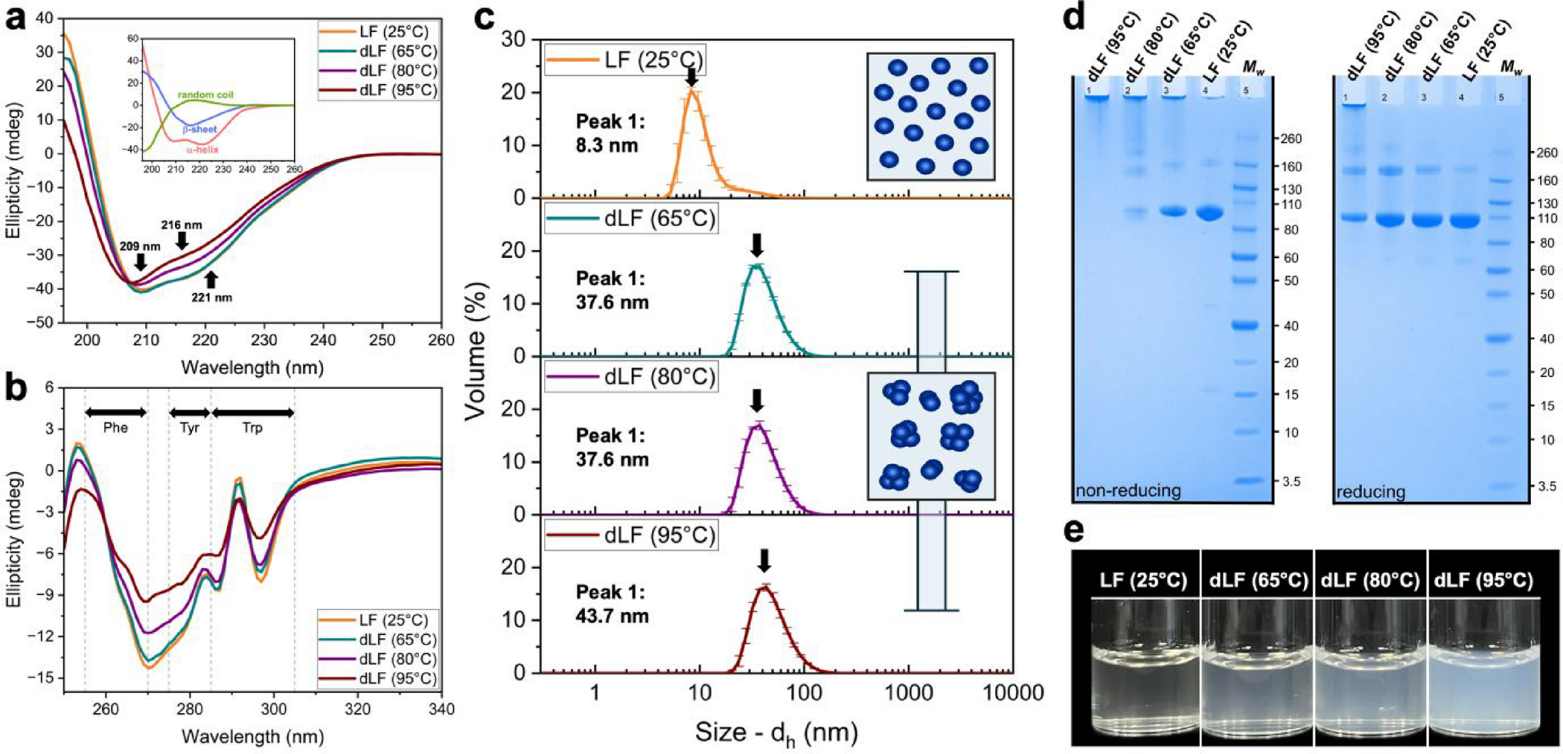
Circular dichroism spectra for native [LF (25 °C)] and heat-treated [dLF (65, 80, or 95 °C)] lactoferrin in HEPES buffer at pH 7.0, showing (a) secondary and (b) tertiary structure changes. Inset in (a): characteristic CD spectra of purely random coil, β-sheet and α-helical protein secondary structures for comparison. (c) Particle size distribution obtained by DLS at 25 °C for LF in HEPES buffer under its native state [LF (25 °C)] or after heat-treatment at 65 °C [dLF (65 °C)], 80 °C [dLF (80 °C)] or 95 °C [dLF (95 °C)]. (d) SDS-PAGE gels of LF and dLF samples under nonreducing and reducing conditions. (e) Macroscopic appearance of 1.0 wt % LF dispersions in HEPES at its native state, or after heat treatment at 65 °C, 80 or 95 °C for 30 min. Samples were not filtered for the picture.

Focusing on tertiary structure, Figure 1b shows the near-UV region where the absorption originates from aromatic residues, and disulfide bonds. For both native LF (25 °C) and denatured dLF (65 °C), the aromatic residues remain largely constrained in specific positions, indicating minimal alterations in tertiary structure between these two samples. The pronounced peaks at 270 and 295 nm suggest that these residues persist in a well-ordered and asymmetric environment, reinforcing the structural stability across these conditions. The characteristic emission of phenylalanine (255–270 nm), tyrosine (275–285 nm), and tryptophan (285–305 nm)^32^ is decreased with increasing denaturation temperatures (Figure 1b), as the signal intensity is attenuated at these characteristic wavelengths. This effect is particularly evident for dLF (95 °C), suggesting significant structural perturbation and a disruption of native interactions. These findings indicate that LF’s tertiary structure undergoes a gradual thermal denaturation process, with partial unfolding observed at 80 °C, and a more pronounced loss of tertiary structure at 95 °C. The retention of some spectral features at elevated temperatures suggests that while tertiary interactions are disrupted, complete unfolding and a compact ‘molten globule’ state may not be achieved under these nonreducing conditions, without a disulfide-bond reducing agent.^33^ For such unfolded state, the residues would adopt multiple conformations, averaging out their contributions and resulting in a near-flat CD spectrum, which does not occur in the case of dLF samples.

Another factor associated with the dampening of the CD signal involves changes in disulfide bond. To determine whether this change in signal was due to disulfide bond rearrangement or complete reduction resulting in free thiol groups, Ellman’s assay was performed to detect the presence of free thiols. For all LF samples, no free thiol groups were detected before or after the heat-treatment at 65, 80 and 95 °C for 30 min (SI, Figure S3). The measured absorbance values for LF with or without heat treatment were below the limit of detection, which agrees with previous results found in the literature.^34^ Thus, the observed changes in near-UV CD spectra reflect structural perturbations around aromatic residues and disulfide linkages, rather than complete disulfide bond cleavage resulting in free sulfhydryl groups. This suggests enhancement of mucoadhesion (if any) by heat treatment of LF cannot be attributed to thiol interactions with cysteine-reactive groups in mucin, often a chemistry probed in the literature for mucoadhesion.

### Thermal Treatment Induces the Aggregation of LF

As a result of structural changes, aggregation, unfolding or a combination of both phenomena may happen upon heat treatment.^14^ Figure 1c shows the DLS results of LF at different temperatures. At 25 °C, the diameter of LF is 8.3 nm as taken from the peak maximum, which agrees with the literature value reported.^35^ Aggregated fractions are also evident at this native state, as a minor secondary population is detected on a high-sized tail which extends up to diameters of 40 nm. Between 56 and 59 °C this tail becomes and independent peak (SI, Figure S4), suggesting some degree of aggregation prior to denaturation. We found that the critical temperature-induced transition in size happens specifically at 60 °C, when the *d*_*H*_ of LF increases from 8.3 to 37.6 nm (SI, Figure S4) and does not increase further when heated to 80 °C (Figure 1c). At 95 °C, holo-LF is also completely denatured, with the peak maximum appearing at 43.7 nm. After cooling the dLF measured at 65, 80, or 95 °C down to 25 °C, the *d*_*H*_ of the denatured LF did not change (Figure 1c shows data obtained at 25 °C for all LF and dLF samples), indicating the irreversible nature of the heat-induced size change after being exposed to temperatures higher than 60 °C.^10^ A higher degree of thermal-induced unfolding leading to aggregation was previously reported for apo-LF when compared to holo-LF,^36^ which may explain why the main difference in size is observed between LF (25 °C) and dLF (65 °C).

The heat denaturation process involves the protein going from a folded, rigid, and compact state (native) to a disordered, flexible, and solvated state (denatured). When the denaturation happens using a reducing agent such as DTT, a molten globule state is achieved.^33^ This state is characterized by local order within a compact structure that retains secondary elements but lacks the long-range organization of tertiary structure, hence the term ‘molten’. Even though we did not probe the interactions of lactoferrin at its molten state with mucin here, it is interesting to ask whether the 4-fold size increase after heat-treatment is due to the protein unfolding, or due to protein aggregation, which may be mediated by disulfide rearrangement. One would expect an increase in the molecular weight of the material if protein aggregation is happening, which can be characterized compositionally using SDS-PAGE in nonreducing conditions (Figure 1d).

Figure 1d shows the molecular weight distribution of LF and dLF measured using SDS-PAGE. For nonreducing conditions, the major protein fraction presented *M*_*w*_ values of LF between 80 and 93 kDa, and a few aggregates with *M*_*w*_ of 150–160 kDa, corresponding to dimers in solution and is in agreement with the literature.^37^ It also contained a few fractions with smaller sizes of 15 and 40–60 kDa. These could be other whey proteins, considered as impurities here, such as α-lactalbumin (14 kDa), β-lactoglobulin (18 kDa – monomer and 36 kDa - dimer), casein (20–27 kDa), or bovine serum albumin (66 kDa). For dLF at 65 and 80 °C, the proportion of proteins weighing 91–93 kDa decreased, and fewer fractions with lower *M*_*w*_ were detected. However, the proportion of aggregates weighing 150 kDa increased. For dLF (95 °C), all monomeric LF disappeared, and new aggregates appeared, with some weighing around 210 kDa and others exceeding 260 kDa. Determining the precise *M*_*w*_ of these larger aggregates is challenging due to limitations in gel resolution and in the molecular weight standard used.

When heating cysteine-containing proteins, one of the mechanisms responsible for protein aggregation following protein unfolding is the formation of new disulfide bonds. Monomeric bovine lactoferrin has 17 disulfide bonds,^12^ which may undergo thiol exchange reactions, facilitating further aggregation. The SDS-PAGE analysis under reducing conditions (Figure 1d) confirms that the observed higher molecular weights for dLF (65 °C) and dLF (80 °C) are partly due to the formation of new disulfide bonds, as their reduction leads to the appearance of lower molecular weight species. For dLF (95 °C), other aggregation mechanisms are likely involved, as aggregates are visible even in the presence of a reducing agent. Figure 1e further confirms the temperature-dependent aggregation, the presence of larger aggregates is evidenced by an increase in sample turbidity. A gradual increase in turbidity was observed among the samples, although all remained macroscopically transparent. This pattern suggests that the dispersions maintained colloidal stability, while also indicating variations in particle size distributions. The LF (25 °C) sample exhibited high transparency and minimal turbidity, consistent with Rayleigh scattering, which arises when particle radii are significantly smaller than the wavelength of visible light. These optical characteristics point to particles within the lower nanometre range. In contrast, the dLF (95 °C) sample displayed higher turbidity and optical density, indicative of a shift toward the Mie scattering regime, which is associated with particles with dimensions approaching the wavelength of light. Although Figure 1c shows that the smallest dispersed units remain similar in size across different dLF samples, the noticeable differences in macroscopic appearance suggest the formation of slightly larger aggregates. These structures, while still within the nanometre scale, likely contribute to enhanced scattering and the observed optical differences.

### Thermal Treatment Induces Changes in the Surface Charge and Hydrophobicity of LF

Zeta-potential measurements confirm that LF is positively charged at pH 7.0 (+20 mV in water and +9.7 mV in HEPES; SI, Table S1), in agreement with previous reports.^10^ The isoelectric point (IEP) value, corresponding to the pH at which the net charge at the hydrodynamic shear plane of the electrical double layer is zero, has been reported to range between 8 and 9 for LF.^12^ This is attributed to the high number of exposed positively charged amino acids (SI, Figure S5). Furthermore, the ζ-potential value increased upon heat-treatment (from +9.7 to +17 mV in HEPES; SI, Table S1). This change is likely related to heat-induced aggregation that modify the shear plane environment. Aggregation can bury negatively charged residues, such as aspartic and glutamic acid, and/or expose positively charged residues, including histidine, lysine, and arginine at the aggregate surface. Besides slight differences in pH, the lower ζ-potentials observed in HEPES buffer (SI, Table S1) are expected owing to the presence of salt, due to a reduction in the Debye length and consequent shrinkage of the electrical double layer. Of more importance, the heat treatment only influences the charge distribution when comparing LF (25 °C) with dLF (65 °C). When comparing denatured LF samples, heat treatment did not influence the charge distribution. In other words, enhancement in mucoadhesion (if any) upon protein denaturation between 65 and 95 °C cannot be attributed to increased surface charge.

As highlighted in the schematic of its primary structure in Figure S5 (SI), LF comprises a significant number of hydrophobic residues. Hence, it was imperative to understand how heat denaturation affects the surface hydrophobicity, which might influence hydrophobic interactions with mucins. Here, surface hydrophobicity was determined by measuring the fluorescent emission at 491 nm after binding LF (25 °C), dLF (65 °C), dLF (80 °C) or dLF (95 °C) to the fluorescent ANS probe (SI, Figure S6). The linear relationship between LF concentration and fluorescence emission at λ = 491 nm after ANS binding reveals a temperature-dependent increase in the surface hydrophobicity index (*H*_0_). Native LF (25 °C) exhibits the lowest *H*_0_ (3.96 × 10^7^). Upon heating to 65 °C, *H*_0_ increases by 62% (6.43 × 10^7^). For dLF at 80 °C, *H*_0_ rises by 182% (1.12 × 10^8^). These increases suggest a progressive structural rearrangement, which is in agreement with the structural trends previously discussed from the data presented in Figure 1a,b. Remarkably, the most pronounced change occurs for dLF at 95 °C, with a 689% increase in *H*_0_ (3.13 × 10^8^) compared to the initial value for LF (25 °C). Previous reports indicate that for smaller heating times (10 min), this increase in surface hydrophobicity is not observed.^38^ While ANS fluorescence may also be influenced by local charge,^39^ the change of the surface hydrophobicity index *H*_0_ for the denatured samples indicates a significant role of hydrophobic exposure, given that the value continues to increase with increasing thermal treatment temperature, while the ζ-potential remained unchanged across 65, 80, and 95 °C.

Atomic Force Microscopy (AFM) images reveal distinct morphological changes in lactoferrin (LF) upon heating, confirming the temperature-dependent structural rearrangements previously described. Native LF (25 °C) (Figure 2a) appears as small, dispersed structures exhibiting relatively low height and full width half-maximum (fwhm) of 6.2 nm, suggesting a compact and stable conformation. For the AFM measurements the LF samples were not filtered, and some larger aggregates are also visible for native LF (Figure 2a,d). It is worth noting that the distribution profile of the aggregated fraction is predominant for dLF (80 and 95 °C) (Figure 2e–g), as larger particle diameters between 10 and 50 nm are noticeable.

**Figure 2:**
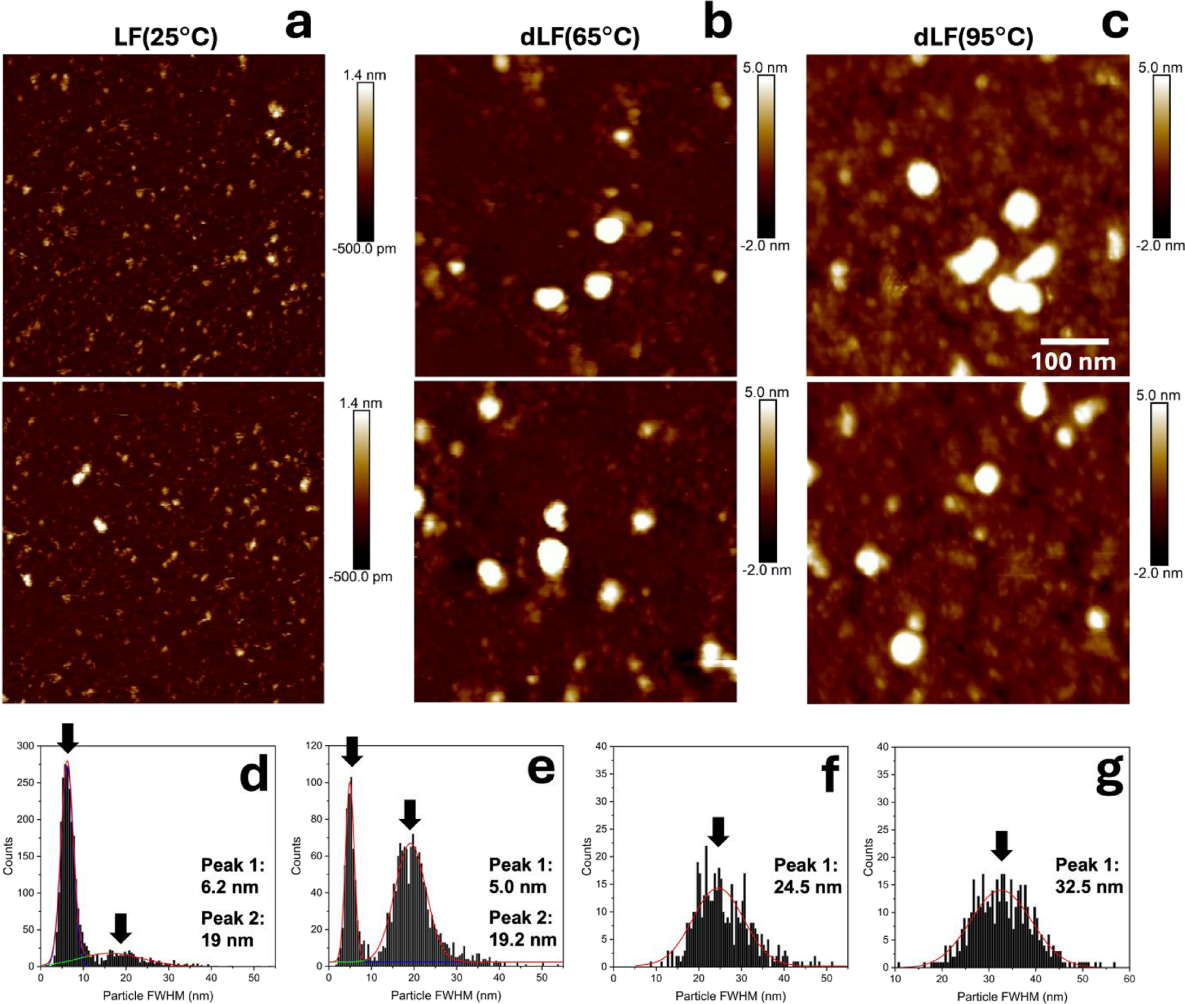
Topographic images obtained using atomic force microscopy (AFM) in the tapping mode for (a) LF (25 °C), (b) dLF (65 °C), and (c) dLF (95 °C) adsorbed onto mica. Scan sizes of 500 × 500 nm are shown together with the histograms showing the full width half-maximum obtained for (d) LF (25 °C), (e) dLF (65 °C), (f) dLF (80 °C), and (g) dLF (95 °C).

While with AFM imaging it is possible to notice increased particle sizes for the aggregated samples, a similar aggregation pattern was observed using DLS (Figure 1c), where increased *d*_*H*_ values are seen. For dLF (65 °C), however, DLS showed a seemingly homogeneous particle size distribution (Figure 1c). In this case, it is plausible that the scattering signal is dominated by the larger particles in solution, whereas the AFM histogram in Figure 2e suggests the presence of two distinct populations. Given that AFM images were obtained for samples that were adsorbed onto mica surfaces and subsequently dehydrated, the absolute size values are not directly comparable to DLS measurements in bulk solution. However, similar observations confirm that higher temperatures promote the restructuring of LF, leading to aggregation. As monomeric structures are no longer visible for dLF at 80 and 95 °C, the increased size and hydrophobicity of aggregates at elevated temperatures may have implications for the functional properties of dLF in various applications, including its interactions with mucin.

To summarize the structural changes on LF, upon heating at 65 °C, DLS reveals that a significant proportion of LF are in an aggregated state. However, CD spectroscopy suggests that most structural features remain comparable between native LF (25 °C) and its denatured counterpart (dLF, 65 °C). As the temperature increases to 80 °C, LF undergoes intermediate denaturation, while at 95 °C, extensive aggregation leads to the loss of detectable monomeric bands in SDS-PAGE and more pronounced structural unfolding in CD. Despite exhibiting similar ζ-potential values, all dLF aggregates display distinct surface hydrophobicity profiles, which may influence their interactions with mucin. Previous studies have reported that for heated and unheated 1.0 wt % LF dispersions, micron-sized aggregates are visible using scanning electron microscopy.^38^ For the next results and discussion section, more concentrated LF and dLF samples are used, and the presence of these aggregates remain evident and will be considered in our discussion. Our ongoing investigations include the use of Differential Dynamic Microscopy to probe the impact of aggregates on LF behavior in solution and on its interaction with mucin, as this technique appears as a robust alternative to DLS for analyzing polydisperse turbid systems.

In the present work, aggregation was investigated as it is relevant in the context of mucoadhesion. Beyond the biomedical relevance of LF-mucin interactions, it is important to note that lactoferrin is a food protein, and aggregation phenomena are central to many of its functional properties.^40^ Protein aggregation in food systems can proceed through different pathways depending on the environmental conditions, with consequences for solubility, stability and texture perception. Although these aspects were not the focus of this work, the mechanistic insights reported here into temperature-induced LF self-association and its interaction with mucin may also contribute to a broader understanding of aggregation pathways relevant to food applications, including texture perception.^41^

### Characteristics of Bovine Submaxillary Mucin

Before probing adhesion of LF and dLF to mucin, it was important to characterize the mucin structure. After the purification protocol, which included dialysis, no minor common protein contaminant fractions such as bovine serum albumin (BSA)^42^ were found in BSM, as shown in Figure 3a. In particular, no protein fraction with *M*_*w*_ lower than 2.6 × 10^5^ g mol^–1^ (maximum resolution of the gel) was detected, indicating that the purified mucinous glycoproteins in BSM have a higher molecular weight than this value. Figure 3b shows the elution profile for BSM using AF4, in which the UV detector (top) and the MALS signal (bottom) were used to calculate the concentration of the eluted fractions, as well as the molar mass (*M*) and radius of gyration (*R*_*g*_). Figure 3c shows the conformation plot arising from the relationship between the *R*_*g*_ of each eluted fraction and *M*. Although the data suggest a consistent scaling behavior across the eluted fraction, the limited molar mass range (1.0 × 10^7^–5.5 × 10^7^ g mol^–1^, i.e., less than 1 order of magnitude) and the slight deviations from linearity limit the reliability of conclusions regarding the presence of a self-similar structure without major conformational transitions in BSM. The red line represents a first-order linear regression fit of the data with a slope that corresponds to the Flory exponent (ν). Typical values for ν and their resulting conformations include 1 for rigid rods, 0.58 for expanded coils in good solvent conditions and 0.33 for collapsed globules in poor solvent conditions.^43^ Here we found ν to be 0.69 ± 0.04 for BSM, suggesting an extended conformation with some degree of chain rigidity, which may be related to electrostatic repulsion between mucin glycosidic groups. However, given the limited *M*_*w*_ range, this exponent needs to be interpreted cautiously, as it may reflect a transition regime between stiff to flexible conformations rather than a true power-law behavior. Previous reports employed small angle light scattering and identified slightly lower values of ν for human airway mucus in the range of 0.36–0.45, which might be related to the presence of other components such as cellular debris in the sample.^44^ In another study on gastric mucins, in which the molecular weight of the samples spanned over 2 orders of magnitude, a difference in stiffness was observed between commercial and native samples, with purified commercial gastric mucins (with *M*_*w*_ between 10^6^ and 10^7^ g mol^–1^) displaying higher rigidity than those purified from native tissues (*M*_*w*_ between 10^6^ and 10^8^ g mol^–1^) which are more flexible.^45^

**Figure 3:**
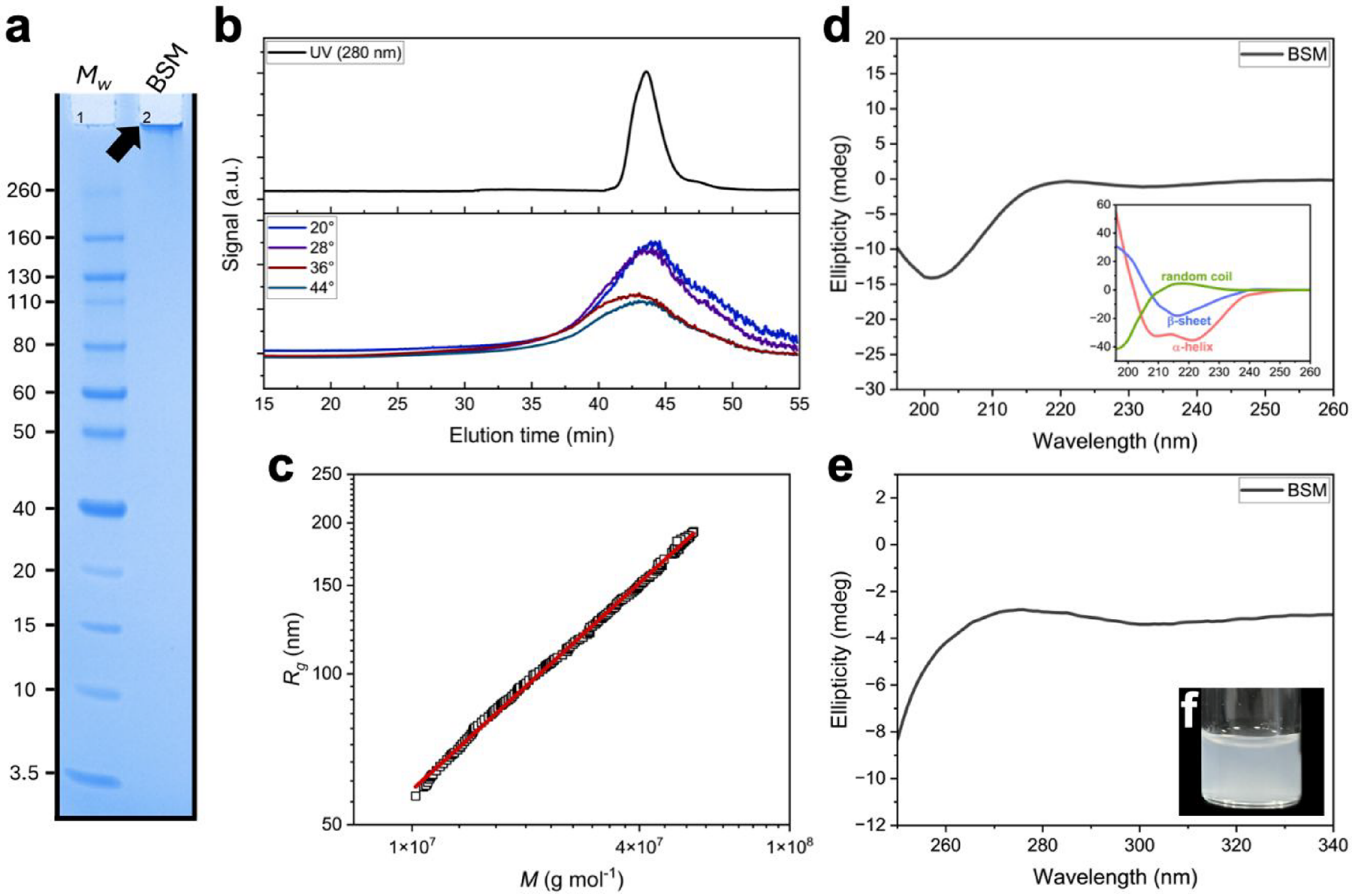
SDS-PAGE gels (a) containing a molecular weight standard (lane 1), and BSM under nonreducing conditions (lane 2). (b) AF4 elution profile of the BSM used in this work, showing the concentration detector signal (UV at 280 nm) and MALS signals at 20°, 28°, 36°, and 44°. (c) Structural conformation plot of the radius of gyration (*R*_g_) versus molar mass (*M*_w_) based on AF4-MALS data, with a solid line representing the first-order linear regression fit of the data with a slope of ν = 0.69 ± 0.04. The CD spectra for BSM show its (d) secondary and (e) tertiary structures. The inset in (d) highlights the characteristic CD spectra of random coil, β-sheet, and α-helical secondary structure elements. (f) Inset showing the macroscopic appearance of a 1 wt % BSM dispersion in HEPES buffer.

Previous reports have identified molecular weights in the range of 10^5^–10^6^ g mol^–1^ for BSM^46,47^ as determined by equilibrium ultracentrifugation and low-angle laser light scattering detectors in high-performance gel chromatography. The dominant mucin type in BSM in MUC5B, for which a single glycosylated polypeptide chain (often referred to as ‘monomer’ in the mucin literature) is expected to have around 3 × 10^6^ g mol^–1^.^48^ However, the formation of oligomers is known for MUC5B, leading to a heterogeneous distribution of molecular weight values,^48^ and the presence of MUC19 and unique peptide sequences has been identified for commercial BSM.^49^ Here, we found molar masses in the rage of 1.0 × 10^7^–5.5 × 10^7^ which is consistent with previous observations of mucin heterogeneity and suggests that the analyzed sample predominantly consist of oligomeric species. Table 1 summarizes the distribution-averaged macromolecular information of BSM.

**Table 1:** Distribution-averaged macromolecular nformation of the BSM used in this work, obtained from AF4-MALS data.

parameter	BSM
*M*_*n*_ (× 10^7^ g mol^–1^)	3.15 ± 0.07
*M*_*w*_ (× 10^7^ g mol^–1^)	3.93 ± 0.12
*Đ* (*M*_*w*_/*M*_*n*_)	1.25 ± 0.05
*R*_*g,z*_ (nm)	165 ± 11
recovery (%)	74.6 ± 2.5

Apomucin, the fully deglycosylated form of mucin, has been estimated to have molecular weights between 5.8 × 10^4^ and 7.0 × 10^5^ g mol^–1^.^50^ These values indicate that approximately 25% of weight consists of amino acid residues in the protein backbone, with the remaining 75% corresponding to glycan moieties.^51^ The steric hindrance and electrostatic repulsion between glycan chains contribute to the adoption of an extended random coil conformation, rather than the compact, folded architecture with α-helical and β-sheet elements typically observed in globular proteins such as LF. The far-UV CD spectrum of BSM (Figure 3d) reveals a small fluctuation in ellipticity near 220 nm and a pronounced negative ellipticity minimum near 200 nm, which are both characteristics of random coil conformations.^42,52^ The absence of well-defined negative bands around 208 and 222 nm suggests minimal α-helical content, while the lack of a strong positive band near 195 nm corroborates the highly flexible nature of the protein.

Figure 3e presents the near-UV CD spectrum of BSM, which exhibits weak ellipticity signal between 260–320 nm. This suggests a lack of highly ordered tertiary packing, in agreement with t he extended and flexible conformation previously reported for mucins.^53^ These results reinforce the view that BSM predominantly adopt an extended random coil-like conformation.

Mucin concentrations in human mucosal environments vary significantly depending on anatomical location, physiological state, and measurement method employed, with reported values ranging from 0.09 wt % in whole saliva^54^ to 6.3 wt % in the large intestine (colonic region).^55^ Based on our measured *M*_*w*_ and *R*_*g*_ values, the estimated critical overlap concentration (c*) for BSM is 0.34 wt % (calculated as $c*∼M_{w}/\frac{4}{3}\pi N_{a}R_{g}^{3}$ ). QCM-D experiments were performed at concentrations below c*, in the dilute regime. Rheological and confocal measurements were conducted at 0.5 wt % (∼0.13 μmol L^–1^), above the overlap concentration but still well below the entanglement regime.

### Effect of Thermal Treatment on Lactoferrin Adsorption to Mucin Measured by Real-Time QCM-D

Quartz crystal microbalance with dissipation monitoring (QCM-D) was employed to investigate the interaction between LF and dLF and BSM, as well as the viscoelastic properties of the resulting hydrated films. All measurements were performed at 25 °C. Prior to the adsorption process, PDMS-coated SiO_2_ sensors were equilibrated with HEPES buffer, as indicated by a stable baseline at Δ*f* ∼ 0. With the coating, all sensors were hydrophobic in character (θ = 109.6° ± 2.5°) before BSM adsorption (inset, Figure 4a). At approximately 30 min, BSM was introduced, inducing a decrease in Δ*f* consistent with mucin layer formation. Following a buffer rinse to remove loosely bound BSM, LF was injected at approximately 100 min. Regardless of the heat treatment employed, all samples readily adsorbed onto the preformed BSM layer, evidenced by a pronounced decrease in Δ*f*. Native LF and dLF (65 °C) showed adsorption at similar levels, dLF (80 °C) showed enhanced adsorption, and dLF (95 °C) showed the greatest adsorption. After buffer rinsing (“B” stage, Figure 4a), Δ*f* increased somewhat, indicating removal of weakly adsorbed LF, though this effect was minimal for dLF (65 °C) and dLF (95 °C). Following the rinse, final frequency shifts indicated increased remaining adsorption for all dLF samples compared to native LF, with similar values for dLF (65 °C) and dLF (80 °C) and the greatest remaining adsorption for dLF (95 °C).

**Figure 4:**
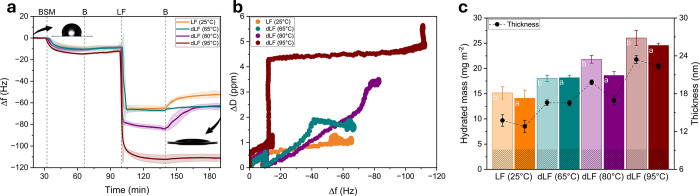
Mean QCM-D frequency shift (a) for the 5th overtone as a function of time, illustrating the sequential adsorption of BSM and LF or dLF samples onto PDMS-coated SiO_2_ sensors. After a stable HEPES baseline (Δ*f* = 0), BSM injection decreased Δ*f* (mucin adsorption), followed by buffer rinsing (“B”). LF samples, either native (orange) or heat-treated at 65 °C (cyan), 80 °C (purple), or 95 °C (red) for 30 min, were then introduced, with final buffer rinsing for all. Shaded areas represent standard deviations from two independent experiments, each using at least three sensors (*n* = 2 × 3). Insets: water contact angle measurements showing hydrophobic sensors prior to adsorption (109.6° ± 2.5°), and hydrophilic surfaces (<20°) after BSM and LF or dLF adsorption. Δ*D* as a function of Δ*f* (b) for each of the LF or dLF samples. Hydrated mass and thickness of BSM-LF/dLF films (c) present at the QCM-D sensor before final rinsing step (columns ‘b’) and after final rinsing with buffer (columns ‘a’); shaded regions correspond to rinsed BSM layers.

Control QCM-D experiments using filtered LF samples (SI, Figure S7) showed no detectable difference in adsorption behavior compared to nonfiltered samples, indicating that pre-existing aggregates in LF did not significantly influence the adsorption onto mucin, either for native or heat-treated LF. These observations confirm that the adsorption behavior captured in Figure 4a primarily reflects the interaction of monomeric or nanometer-sized LF aggregates with the mucin layer. In QCM-D in addition to changes in frequency, changes to the dissipation factor can also be measured and enable assessment of the viscoelastic properties of the adsorbed films. To account for the viscoelastic nature of the adsorbed films (Figure 4b), the Voinova model was applied. This assumes that the film behaves mechanically as a Voight model, i.e. a combination of an elastic spring and a viscous dashpot in parallel, and provides predictions for the shifts in frequency Δ*f* and dissipation factor Δ*D* across multiple harmonics.^22^ By plotting Δ*D* against Δ*f*, the slope of the resulting curve serves as a qualitative indicator of the viscoelastic character of the film, where steeper slopes indicate more dissipative and less rigid layers.^56^ Here, the Δ*D*/Δ*f* plots (Figure 4b) revealed distinct final structures among samples: native LF and LF treated at 65 °C exhibited relatively compact and rigid layer formation (lower Δ*D* per unit Δ*f*), whereas LF treated at 80 °C and especially at 95 °C formed increasingly viscoelastic films with enhanced dissipative properties, indicative of more hydrated and flexible structures. This enhanced *soft* character is also evidenced by the spread distribution of frequency across different harmonics (SI, Figures S6 and S7).

The hydrated mass derived from QCM-D measurements is model-dependent, as the film mass contribution and the layer thickness are correlated (*h*_layer_ρ_layer_ω), therefore, the calculated output is a composite parameter. With the thickness value calculated from the dissipation values, another relevant parameter is the density. To use equation 3) to estimate the hydrated mass, a range of plausible layer densities was considered, from 1006 g/L (buffer density, no LF or dLF adsorption) to 1400 g/L (for a denser protein layer). Across this range, the estimated thickness of dLF (95 °C) was found to vary between 22 and 24 nm, corresponding to a 10% variation, while the corresponding hydrated mass varied by 15%. Despite the influence of hydration on the absolute values of mass and thickness, the observed trends in Figure 4 cannot be fully explained by hydration alone. Increased mass and thickness, particularly for dLF (95 °C), suggest enhanced adsorption of thermally denatured LF onto the mucin layer, likely due to the exposure of hydrophobic domains upon structural unfolding (Figures 1 and S5). Although all dLF samples showed similar size (Figure 1) and charge properties (Table S1), heating dLF to 95 °C resulted in a 386% increase in surface hydrophobicity and a corresponding 35% increase in hydrated mass in QCM-D experiments when compared to dLF (65 °C).

While hydration effects may contribute to the QCM-D-derived mass and thickness, the enhanced interaction between heat-treated LF and mucin is primarily attributed to thermally induced structural changes, which may facilitate increased hydrophobic interactions.

### Heat-Induced Network Formation in Lactoferrin–Mucin Complexes

Confocal laser scanning microscopy (CLSM) was employed to characterize the microstructure of complexes formed between LF or dLF with BSM. LF was fluorescently labeled with fast green, which emits in the red channel, while BSM was stained with calcofluor white, emitting in the blue channel. In control LF dispersions stained with both dyes, fluorescence was predominantly observed in the red channel, consistent with the limited glycosylation of LF, which results in negligible signal from calcofluor white (SI, Figure S9). This outcome is in agreement with the known structure of LF, which contains relatively few glycosylation sites (SI, Figure S5). In contrast, CLSM analysis of BSM which is characterized by extensive glycosylation, revealed fluorescence in both channels, due to the binding of fast green to proteinaceous domains and calcofluor white to carbohydrate residues^57^ (SI, Figure S9).

The microstructure of LF/BSM mixtures was found to be heterogeneous, with dispersed particles comprised of colocalized proteinaceous and carbohydrate-rich regions. Notably, varying the LF:BSM ratio did not result in significant qualitative changes to the microstructural organization, although somewhat smaller particle sizes with increased number density were observed at higher LF concentrations (SI, Figure S10). The colocalization of the two components, with no noticeable regions of LF alone, suggests specific interactions, likely driven by electrostatic forces between the positively charged LF (+9.7 mV; SI, Table S1) and the negatively charged mucin at pH 7.0 (−25.5 mV; SI, Table S1). Previous reports on the self-assembly of oppositely charged proteins were comprehensively reviewed Bouhallab and Croguennec.^58^ These studies demonstrate that electrostatic association depends strongly on the pH relative to the isoelectric point of the proteins involved. While this analogy applies for native LF, a key distinction is needed for mucins, as in their case the overall negative charge arises from terminal glycans, rather than from peptide side chains. Unlike the relatively rigid and folded structure of LF, the highly glycosylated mucins are conformationally flexible and solvent-exposed,^59^ features that influence both the strength and specificity of their electrostatic interactions with positively charged proteins such as LF.

Considering the impact of heat-treatment, LF (25 °C) and dLF (65 °C) formed similar colloidal structures upon mixing with BSM, exhibiting no appreciable differences in aggregation profile (Figure 5). Strikingly, upon thermal denaturation of LF at the elevated temperatures of 80 or 95 °C, the resulting BSM/dLF (80 °C) or dLF (95 °C) complexes displayed a network-like microstructure observable in both individual and composite CLSM images, instead of the dispersed aggregates previously seen for LF (25 °C) and dLF (65 °C) (Figure 5). These two mixtures with distinctive microstructure also displayed similar response to shearing conditions, as the next section shows. These findings imply that treatment at elevated temperatures (80 and 95 °C) enhances LF/BSM association, primarily through increased hydrophobic interactions facilitated by conformational unfolding of LF (SI, Figure S6). Since no free thiol groups were detected in either native or denatured LF as previously discussed, any possibility of disulfide bond exchange would necessarily rely on mucin-derived thiols. Under the neutral (pH 7.0) and nonreducing conditions used, where only a small fraction of thiols exist as reactive thiolates and LF protein structure provide intrinsic stability to its disulfides, the possibility of covalent cross-linking with cysteine reactive groups in mucin is limited. As shown in Figure 1b, incubation at 80 and 95 °C led to marked disruptions in the tertiary structure of LF, which is consistent with increased exposure of reactive hydrophobic domains.

**Figure 5:**
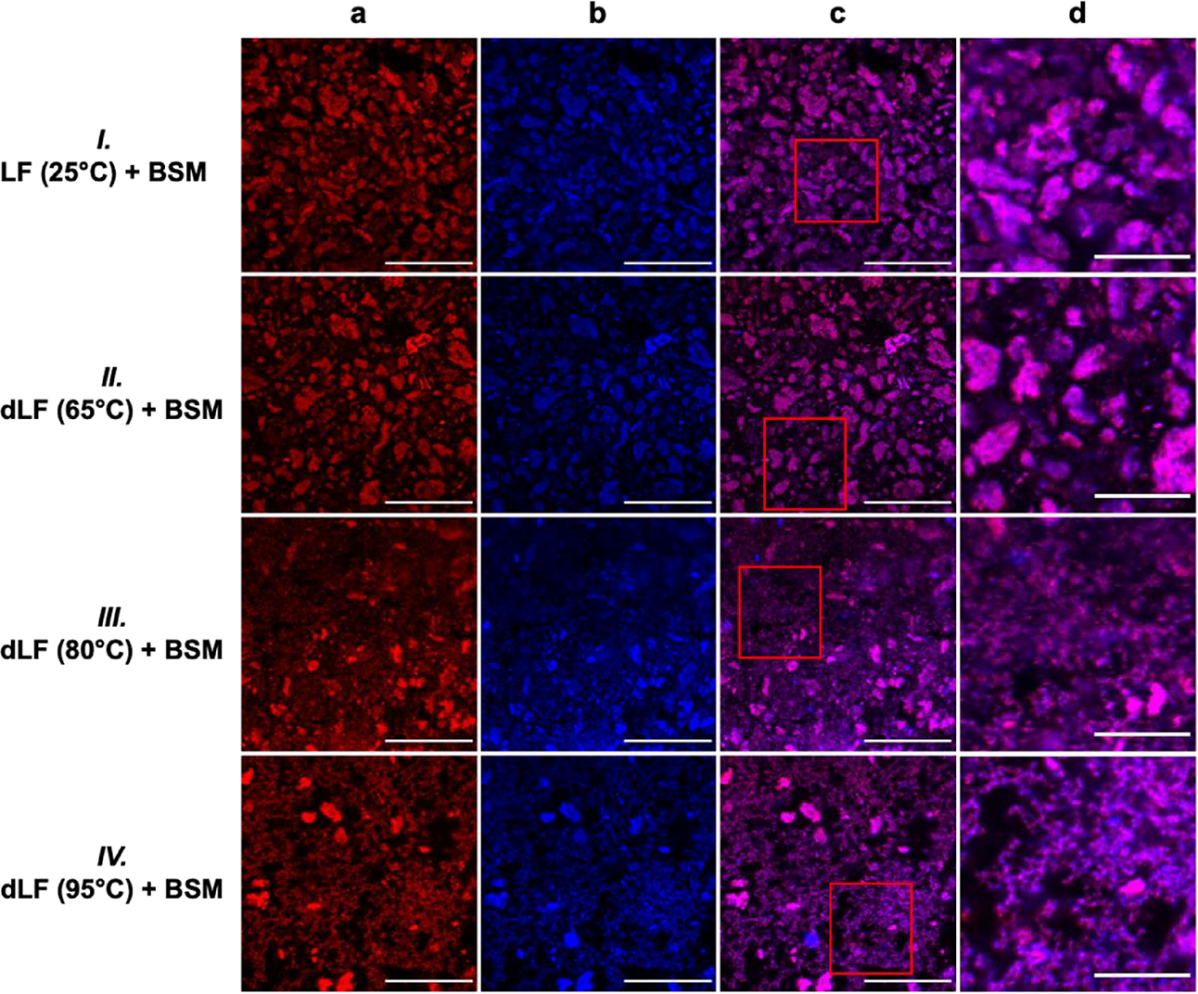
Confocal laser scanning microscopy (CLSM) images of complexes formed between bovine submaxillary mucin (BSM) and lactoferrin (LF) or thermally denatured lactoferrin (dLF) at a concentration of 0.5 wt % for each component. Samples correspond to (I) BSM/LF (25 °C), (II) BSM/dLF (65 °C), (III) BSM/dLF (80 °C), and (IV) BSM/dLF (95 °C). Panel (a) displays the fast green channel (proteinaceous structures) excited at 633 nm, panel (b) shows the calcofluor white channel (carbohydrate-rich structures) excited at 360 nm, panel (c) presents the merged composite image, and panel (d) provides a magnified view of the region highlighted in red in (c). The scale bar corresponds to 50 μm in images (a–c), and to 20 μm in image (d).

### Lactoferrin–Mucin Binding Promotes Viscosity Enhancement as Measured by Shear Rheology

Following the in-depth characterization of LF, dLF and BSM, we probed the shear rheological response with the hypothesis that increased mucoadhesion by LF or dLF will increase viscosity particularly at larger shear rates where the shear viscosity plateau is approached. The shear response of LF and BSM at 0.5 wt % each, both individually and in mixtures, was investigated using a double-gap (DG) geometry. Using this geometry, the viscosity of the buffer, which is equal to that of pure water, can be measured at the reported range of shear rates (Figure 6). The steady-shear apparent viscosity η­(γ̇) as a function of shear rate (γ̇) was determined to evaluate potential interactions and the influence of thermal treatment on LF (Figure 6a–d). The measured viscosity data suggest that LF solutions are shear-thinning for all tested conditions. However, the reasons for this behavior require careful consideration, since the flow curves in Figure 6a–d for the pure LF and dLF components show pronounced shear-thinning behavior at concentrations as low as ϕ = 0.01, which is a much lower concentration than expected (∼62.5 μmol L^–1^). In contrast, 125 nm latex particles (nearly hard spheres) do not show shear-thinning behavior until a volume fraction ϕ = 0.2.^60^ Moreover, when measuring the stress buildup response after applying a preshearing condition of 100 s^–1^, a 0.5 wt % dispersion of LF (25 °C) took up to 300 s to reach steady state conditions at lower shear rates (<5 s^–1^) (SI, Figure S11). Additionally, much longer equilibrating times are needed if the sample is not presheared (besides the inevitable preshearing from the sample loading into the geometry, which we estimate being between 7 and 40 s^–1^ when using a standard plastic 3 mL Pasteur pipet). It is possible that the observed behavior does not originate from the changes to the bulk rheology, but is instead a consequence of interfacial phenomena, in which protein migrates from the bulk and adsorbs at the liquid/air interface. Previous reports showed a similar rheopectic behavior (stress increase with time in steady shear) for bovine serum albumin (BSA) and bovine synovial fluid.^61^ It is known that upon adsorption at the liquid–air interface, LF structurally unfolds.^62^ Although there are no previous reports of this interfering with LF’s rheological response, similar shear-thinning behavior arising from interfacial effects for milk proteins, such as BSA has been reported previously.^63,64^ In these cases, protein adsorption at the air–water interface resulted in the formation of a viscoelastic film, subsequently influencing the torque readings of the rheometer, even when using geometries with high bulk-to-surface area ratio such as the DG geometry used here.

**Figure 6:**
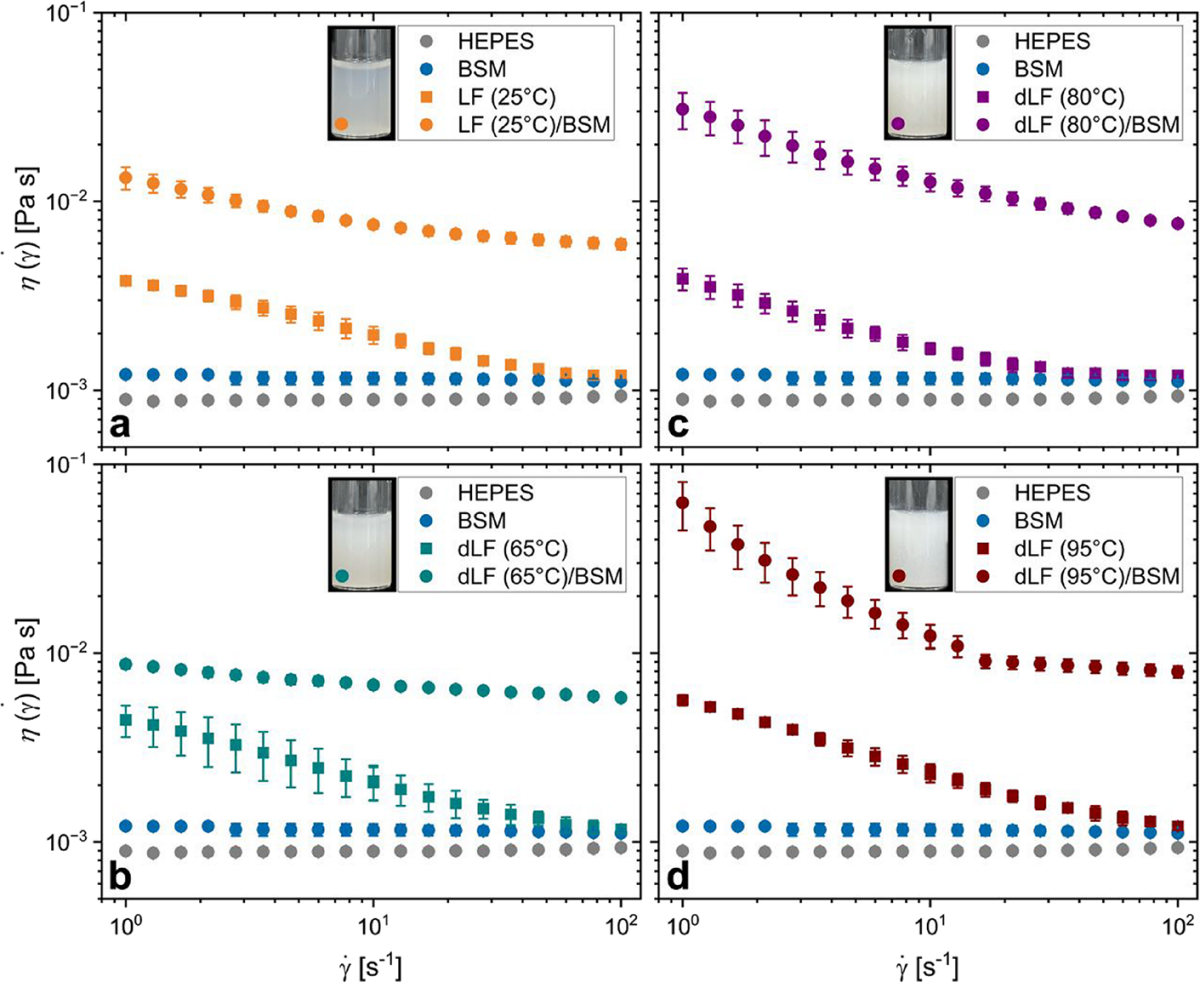
Steady-shear viscosity as a function of shear rate measured using the double-gap (DG) geometry for BSM, HEPES buffer and LF or dLF on their own and their respective mixtures with BSM: (a) LF (25 °C), (b) dLF (65 °C), (c) dLF (80 °C), and dLF (95 °C). Each component (LF, dLF or BSM) is present at 0.5 wt % each. An inset in each (a–d) graph shows the macroscopic appearance of the mixtures of BSM and LF analyzed in the rheometer: (a) BSM+LF (25 °C), (b) BSM+dLF (65 °C), (c) BSM+dLF (80 °C), and (d) BSM+dLF (95 °C).

When steady-state viscosity measurements were conducted with cone-and-plate geometries, which presents a significantly higher surface-to-volume ratio, enhanced contribution of interfacial effects led to even higher measured viscosity values for LF and dLF samples (SI, Figure S12). The Boussinesq number (*Bo*),

$$Bo=\frac{\left(\eta_{s}v/L_{s}\right)P_{s}}{\left(\eta v/L_{b}\right)A_{b}}=\frac{\eta_{s}}{{\eta l}_{s}}$$ 5

quantifies the contribution of interfacial stresses relative to bulk viscosity and can aid in understanding the interfacial effects in the measurements using each geometry. In eq 5, η_*s*_ is the interfacial viscosity, *v* is the characteristic velocity (m s^–1^), *L*_*s*_ and *L*_*b*_ are the length scales for the shear flow in the interface and at the bulk, *P*_*s*_ is the contact perimeter between the interface and the geometry (m), *A*_*b*_ is the contact area between the geometry and the bulk (m^2^), and *l*_*s*_ = *A*_*B*_/*P*_*s*_ representing a characteristic length scale for each geometry.^63^ The Boussinesq number depends strongly on two parameters: *l*_*s*_ and on the ratio between η_*s*_ and η. Even though Bo is approximately 3 times greater for the CP geometry compared to the DG geometry (SI, Table S2), the ultimate confirmation on whether interfacial effects can be safely neglected depends also on *n*_*s*_/η, which in turn depends on the shear rate. The interfacial behavior of LF was confirmed by an interfacial test using the bicone geometry placed at the liquid/air interface, which identified a higher *G*′ than *G*″ for the viscoelastic film formed (SI, Figure S13), with *G*′ > *G*″ in oscillatory sweeps and a shear-thinning response up to 10 s^–1^. A full investigation on the interfacial behavior of LF and dLF and their resulting complexes with BSM was not possible due to insufficient material; however, from the LF response it is possible to gain insights into at which shear rates it is safe to assume that the bulk response dominates over interfacial effects. Ultimately, bulk flow is negligible if |*Bo*| ≫ 1; while interfacial effects are negligible provided that |*Bo*| ≪ 1. The fact that *Bo* ≫ 1 at a shear rate of 1 s^–1^ (SI, Table S2) suggest that the shear-thinning response of LF at low shear rates is largely an experimental artifact rather than an intrinsic material bulk property. However, since Bo is 0.8 (SI, Table S2) at 100 s^–1^ these effects are avoided and the contribution from the surface is much smaller when using the DG.

Complete avoidance of the interfacial contribution to the torque readings was obtained by introducing dilute solutions of the surfactant Triton X-100 (TRX-100) to the liquid–air interface, at final concentrations of 0.5 wt % LF or dLF and 0.001 wt % of TRX-100. These are known to disrupt protein adsorption at air–water interfaces by reducing surface tension and competing for interfacial space.^65^ Upon addition of TRX-100, the shear-thinning behavior of LF disappeared, and the sample exhibited a Newtonian response, further exemplifying the role of interfacial effects in the observed rheology (SI, Figure S14a). However, surfactant solutions could not be employed in obtaining the bulk viscosity of LF/BSM mixtures, as TRX-100 interacts heavily with mucin, leading to complex formation and precipitation, impeding meaningful rheological measurements (SI, Figure S14b). Surfactants are known to disrupt hydrophobic cross-links in mucin,^66^ and phase separation has been previously observed in three-component mixtures of mucin, Tween80 and the cationic surfactant tetradecyltrimethylammonium chloride.^67^

When LF or dLF were mixed with BSM at equal weight fractions (0.5 wt % each), the resulting η values revealed significant deviations from the individual component behaviors. Even taking account of the interfacial contributions, the viscosity of LF/BSM mixtures exceeded what would be expected from the viscosity of the individual components, evidencing interactions between the components at all shear rates. An order of magnitude increase in the viscosity is observed when looking at the high-shear rate limit, for instance, where bulk contributions predominate (Figure 6a). The influence of LF thermal treatment on these interactions is also noteworthy (Figure 6b–d). LF subjected to 95 °C treatment exhibited the highest viscosity when complexed with BSM, with a 1.4-fold increase was observed in the high-shear rate limit when comparing it to LF (25 °C)/BSM; dLF (80 °C)/BSM exhibited similar high-shear viscosity to dLF (95 °C), while that of dLF (65 °C)/BSM was essentially unchanged compared to LF (25 °C)/BSM. This suggests that structural modifications induced at the elevated dLF treatment temperatures (80 and 95 °C) enhanced its associative interactions with mucin. As discussed previously, heat treatment induces partial unfolding in LF, exposing hydrophobic domains which are newly accessible binding sites facilitating stronger interactions with mucin glycoproteins. Macroscopic observations of LF/BSM mixtures (inset, Figure 6a–d) further corroborate these findings. Phase separation was not evident in any of the tested conditions, implying sufficient intermolecular repulsion that maintain dispersion stability. Previous reports on mixtures of two oppositely charged proteins, LF and β-lactoglobulin, showed that the two liquid protein solutions complexed into a coacervate (complex coacervation) with an exceptionally high viscosity.^68^ In contrast to this phase-separated system, stable mixtures were obtained when mixing LF and the anionic polysaccharides carrageenan and xanthan gum.^69^ Here, when complexation occurs between positively charged LF or dLF and mucin glycoproteins, complex coacervation is not observed. Variations in sample opacity (Figure 6) suggest that structural rearrangements or aggregation phenomena occur to differing extents depending on the thermal treatment of LF, which were previously confirmed using confocal microscopy. Interestingly, the two interconnected microstructures for BSM complexed with dLF (80 °C) and dLF (95 °C) previously shown in Figure 5 respond similarly to shearing conditions at 100 s^–1^.

The schematic representation in Figure 7 illustrates how protein adsorption at the air–water interface can generate artificial shear-thinning behavior in bulk rheological measurements. Given the relatively low concentration of LF on its own used in this study, the formation of an interconnected network within the bulk is unlikely, even if long-range interparticle forces are evoked. Previously, the shear rheological response of LF has been reported to depend on the iron saturation level, as holo-LF presented a higher degree of shear-thinning than apo- and native-LF.^10^ Here, we highlight that this may be related to the interplay between interfacial activity interfering in bulk viscosity measurements; therefore, interfacial contributions need to be considered when interpreting protein solution behavior. Overall, this interplay remains a crucial aspect in understanding the properties of LF-mucin systems, particularly in physiological contexts.

**Figure 7:**
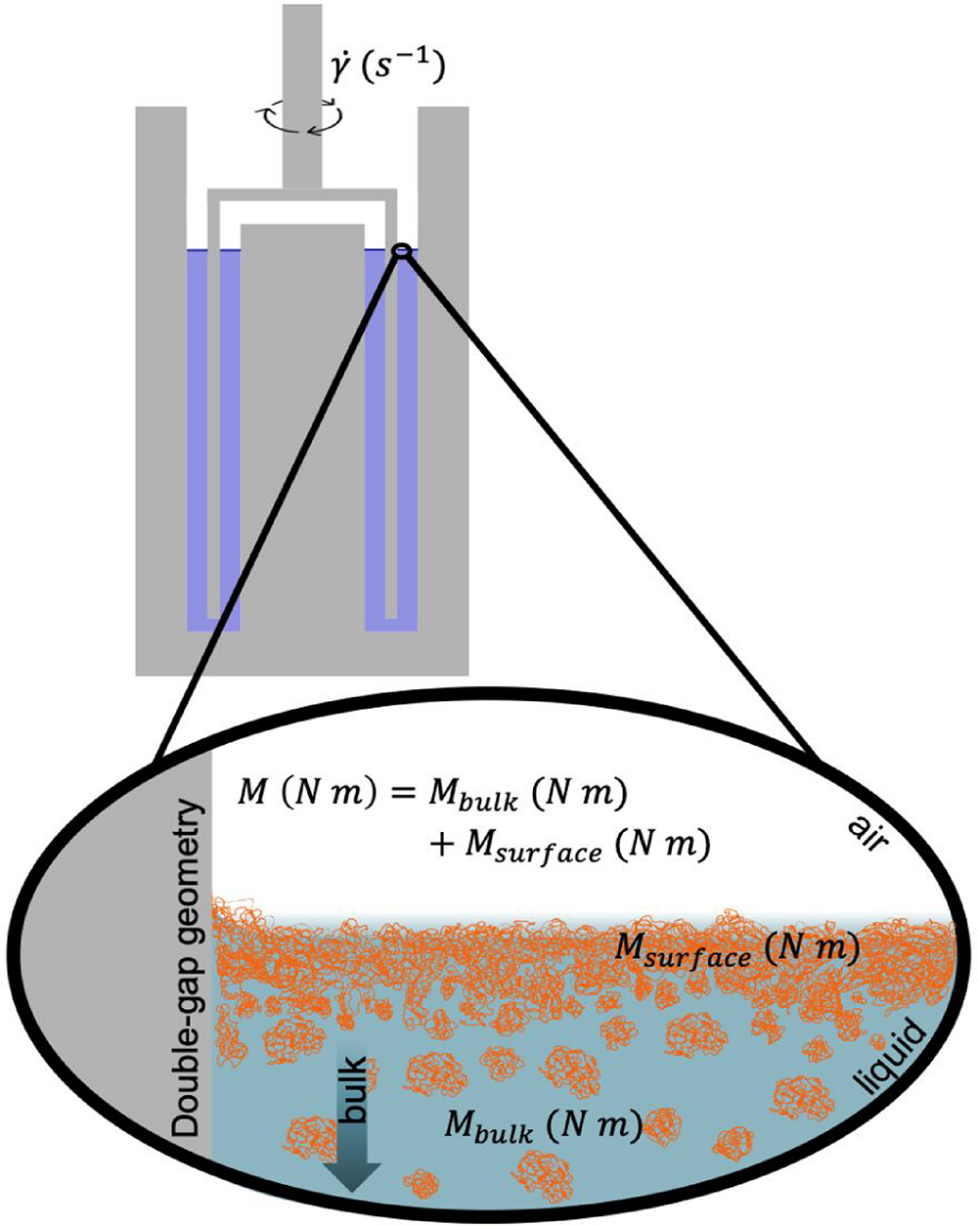
Schematic illustration of LF’s adsorption at the liquid/air interface which interferes in the generated data, as an artificial shear-thinning behavior is observed due to the surface (*M*_surface_) contribution to the total torque (*M*) measured.

## 4 Conclusions

This work advances our understanding of how thermally induced structural modifications in lactoferrin, a food protein, modulate its interaction with mucin, offering molecular insights for designing new protein-based mucoadhesive systems. DLS and AFM showed native LF monomers progressively aggregating with increasing temperature, supported by an increase in visual turbidity, and with denaturing gel electrophoresis showing increasing molecular weights and loss of monomers. No increase in ζ-Potential-potential was found between the denatured forms of LF (hence not a factor in muco-adhesion) but a fluorescence assay showed the hydrophobicity increased markedly with denaturation, to an additional 63% at 65 °C, 182% at 80 °C, and a significant 689% at 95 °C. The LF/dLF – mucin binding was then tested using a bovine submaxillary mucin, which adopted an extended random coil conformation and 165 nm radius of gyration. It was found the increasing LF denaturation enhanced mucin-binding, as evidenced by increased viscosity in mucin-LF/dLF mixtures, the formation of mucin-dLF (95 °C) networks and increased adsorption to mucin films using QCM-D. We highlight that the interfacial behavior of lactoferrin can interfere with rheology measurements, and must be eliminated or carefully considered when interpreting macrorheological data for mucin–protein dispersions. Despite these interfacial effects, we show that heat-treated lactoferrin at *T* > 80 °C exhibited increased viscosity in mucin–lactoferrin complexes greater than the combined individual components, consistent with the formation of networks as observed by confocal microscopy. Quartz crystal microbalance with dissipation monitoring further confirmed a 1.7-fold enhanced adsorption of dLF (95 °C) compared to LF (25 °C) onto mucin surfaces, and structural differences where the native LF and dLF (65 °C) films on mucin were relatively compact and rigid, but increasingly viscoelastic and hydrated with dLF (80 and 95 °C).

Collectively, these findings suggest that modulating protein conformation via thermal processing is a promising route for tailoring mucoadhesive properties in food or biomedical applications. Proteins which exhibit controlled assembly may be ideal candidates for mucosal adhesive systems. However, it should be noted that the mucin used in this work differs from the heterogeneous mucosal environment in vivo, and more research into these systems is necessary.

In the context of practical applications, this study emphasized that the design of mucoadhesive protein systems should not rely solely on native physicochemical properties, such as the isoelectric point, but rather consider how the dynamic structure can be thermally tuned to drive interactions with mucins, offering new insights into the rational design of food protein-based mucoadhesive systems for biomedical applications.

## Supplementary Material



## Notes

Circular dichroic (CD) spectra for LF samples in water versus in HEPES buffer; CD experimental data and corresponding fits; calibration curve for thiol quantification using l-cysteine (l-cys) standards; detailed size-volume volume distribution obtained by DLS at 25 °C for 0.01 wt % LF transitioning to dLF; schematic representation of the structure of bovine (*Bos taurus*) lactoferrin; determination of the surface hydrophobicity index for LF and dLF samples; raw data for the QCM-D frequency shift of different overtones using filtered LF or dLF samples; frequency shift raw data of one QCM-D sensor for each LF or dLF sample; CLSM images of LF and BSM on their own or in mixtures with increasing LF concentration; transient steady shear rheological behavior of LF (25 °C); viscosity of LF or dLF and LF or dLF/BSM samples using a cone and plate geometry; liquid–air interfacial characterization of LF (25 °C); steady shear viscosity of LF and dLF samples in the presence of a surfactant; ζ-potential values for LF or dLF samples in water and in HEPES buffer; and Boussinesq number for the double gap and cone and plate geometries (PDF)
