# Supplementary material for: Heat-Induced Structural Changes in Lactoferrin for Enhanced Mucoadhesion
Source: ACS Appl Bio Mater. 2025 Oct 18;8(11):10255–71. doi: 10.1021/acsabm.5c01534 (PMC12628292; doi:10.1021/acsabm.5c01534)
Supplement: Supplementary file 1 [file mt5c01534_si_001.pdf]

Supporting information

# Heat-induced structural changes in lactoferrin for enhanced mucoadhesion

*Bianca Hazt [a], Daniel J. Read [b], Oliver G. Harlen [b], Wilson C.K. Poon [c], Adam O'Connell [d], Simon D. Connell [e], Anwesha Sarkar [a]**

[a] Food Colloids and Bioprocessing Group, School of Food Science and Nutrition, University of Leeds, Leeds LS2 9JT, UK

[b] School of Mathematics, University of Leeds, Leeds LS2 9JT, UK

[c] School of Physics and Astronomy, University of Edinburgh, Edinburgh EH9 3FD, UK

[d] Polymer Science Platform, Reckitt Benckiser Healthcare (UK) Ltd, Hull HU8 7DS, UK

[e] Molecular and Nanoscale Physics Group, School of Physics and Astronomy, University of Leeds, Leeds LS2 9JT, UK

E-mail: A.Sarkar@leeds.ac.uk

Number of pages: 21

Number of figures: 14

Number of tables: 2

Figure S1 – Circular dichroism (CD) spectra for lactoferrin (LF) samples in water *versus* 10 mM HEPES buffer at pH 7.0 for (a) LF (25 °C) and (b) denatured lactoferrin (dLF) (80 °C), with black arrows indicating characteristic peak values for $\alpha$-helical and $\beta$-sheet structures and grey arrows indicating the shift caused by HEPES buffer. Data are reported as means of three replicates for each of two independent samples measured on separate days (n = 3 × 2).

Figure S2 – CD experimental data and corresponding fits obtained using BeStSel to determine the secondary structure components of (a) LF (25 °C), (b) dLF (65 °C), (c) dLF (80 °C), (d) dLF (95 °C). The structural composition based on each fit for LF or dLF is represented in (e).

Figure S3– Calibration curve for thiol quantification using L-cysteine (L-cys) standards. The absorbance at 412 nm was plotted against L-cysteine concentration, yielding a linear fit with the equation $Absorbance = 0.11396 + 1.34981[Thiol\ concentration]$ and coefficient of determination $R^2$ of 0.99721. For LF and heat-treated LF at 65 °C, 80 °C or 95 °C for 30 min [dLF], no free thiol groups were detected as all absorbance values were below the limit of detection (LOD) of 0.0035 mmol $L^{-1}$. For BSM, an absorbance of 0.31 was read, corresponding to 0.15 mmol $L^{-1}$ of thiol groups. BSM has molecular weights ranging between $1 \, x \, 10^7$ to $5 \, x \, 10^7$ g/mol determined using AF4 (see Figure 3c), and considering a glycosylation contribution of 50-80% on the molecular weight yields 30 - 375 thiol groups per mucin molecule, which is in agreement with the 262 cysteine residues found in bovine MUC5B (UniProt ID F2FB42)[1]. Each sample/standard was read in triplicate for three different samples (n = 3 x 3) and results are reported as mean ± standard deviation.

Figure S4 – Particle size distribution obtained by dynamic light scattering (DLS) at 25°C for 0.01 wt% LF in 10 mM HEPES buffer at pH 7.0 showing the sudden transition between 59 and 60 °C when all monomer sizes of LF disappear and are no longer observed after 60 °C, indicating protein denaturation and irreversible aggregation. Data represents means ± standard deviation of two replicates measured in triplicate (n = 3 × 2).

Figure S5 – Schematic representation of the primary structure of bovine (*Bos taurus*) lactoferrin, drawn based on the UniProt entry P24627[2]. The amino acid numbers for signal peptides are 1 to 19, of which one is a cysteine. Without the signal peptides, bovine lactoferrin contains 689 amino acids. In this representation, each small circle accounts for one amino acid, and these amino acids can be classified into different groups following the properties of their side chain. Here, we adopted this classification[3] to group the amino acids based on their polarity (hence yielding two classes – polar and nonpolar or hydrophobic amino acids) and charge (positively and negatively charged amino acids). The sulfur-containing amino acids are here separated in two different categories – "polar and Sulfur-containing" and "hydrophobic and Sulfur-containing", given the property of free thiols from cysteine groups to interact with mucin. The potential glycosylation sites are typically occupied by *N*-linked glycans and if the 19 signal-sequence amino acids are not shown, the glycosylated asparagine sites are 233, 281, 368, 478, and 547[4]. The one and three-letter symbols and names for the amino acids represented are: A – alanine – ala, C – cysteine – cys, D – aspartic acid – asp, E – glutamic acid – glu, F – phenylalanine – phe, G – glycine – gly, H – histidine – his, I – isoleucine – ile, K – lysine – lys, L – leucine – leu, M – methionine – met, N – asparagine – asn, P – proline – pro, Q – glutamine – gln, R – arginine – arg, S – serine – ser, T – threonine – thr, V – valine – val, W – tryptophan – trp, Y – tyrosine – tyr. The color-coded 3D representation based on each amino acid character was generated using ChimeraX[5].

Figure S6 – Linear relationship observed between different concentrations of LF and their respective fluorescence emission measured at $\lambda$=491 nm after reacting unheated (a) or heat-treated LF at 65, 80 or 95°C (b), (c), and (d), respectively, with the fluorescent probe 8-Anilinonaphthalene-1-sulfonic acid (ANS). The angular coefficient of each linear regression corresponds to the surface hydrophobicity index ($H_0$) of each

sample, as discussed in the main text. Measurements were performed in triplicate for three samples (n = 3 × 3) and results are reported as mean ± standard deviation for all nine readings.

Figure S7 – Quartz crystal microbalance with dissipation monitoring (QCM-D) showing the frequency shift for the 5th, 7th and 9th overtones as a function of time, illustrating the sequential adsorption of bovine submaxillary mucin (BSM) and filtered lactoferrin (LF) samples onto PDMS-coated $SiO_2$ sensors. A stable baseline at $\Delta f = 0$ is obtained for HEPES buffer. Then, a BSM dispersion is injected at ~2000s in the QCM-D chamber, which leads to a decrease in $\Delta f$ indicating mucin adsorption, followed by rinsing with HEPES buffer at ~4000s. Subsequently, filtered LF samples (by 0.22 $\mu$m hydrophilic membranes), either unheated LF (25 °C) or heat-treated, dLF (80 °C) for 30 min, were introduced. The last solution added was HEPES buffer at ~8280s.

Figure S8 – QCM-D frequency shift showing raw data for one sensor each for (a) untreated LF (25 °C) or heat-treated (b) dLF (65 °C), (c) dLF (80 °C), or (d) dLF (95 °C) for 30 min, showing overtones from the 3rd (f3) to the 13th (f13) as a function of time. BSM dispersions were injected at ~2000s in the QCM-D chamber, followed by rinsing with HEPES buffer at ~4000s. Subsequently, LF or dLF samples were introduced at 6000s. The last solution added was HEPES buffer at ~8280s.

Figure S9 – Confocal micrographs of LF *I.* and BSM *II.* at 0.5 wt%. Each LF or BSM was prepared in the presence of 200 ppm of calcofluor white and 200 ppm of fast green, excited at 360 nm and 633 nm, respectively. (a) Transmitted light channel (b) fast green channel (proteinaceous structures) (c) calcofluor white channel (glycan structures) (d) composite of the fast green and calcofluor white channels. The white scale bar in each image represents 50 $\mu$m. Images were obtained with a 40x oil-immersed objective.

Figure S10 – Confocal micrographs of BSM/LF (25 °C) mixtures with 0.5 wt% BSM and *I.* LF at 4.0 wt% (highest concentration), *II.* LF at 2.5 wt%, and *III.* LF at 1.0 wt% (lowest concentration). Both LF and BSM were prepared in the presence of the staining agent - 200 ppm of fast green in the case of LF and 200 ppm of calcofluor white in the case of BSM, which were excited at 633 and 360 nm, respectively. The white scale bar in each image represents 50 $\mu$m. Images were obtained with a 40x oil-immersed objective, and the channels represented are (a) transmitted light, (b) fast green, (c) calcofluor white, (d) fast green and calcofluor white composite.

Figure S11 - Time-dependent rheological behavior of LF (25 °C) under steady shearing conditions, following a pre-shearing condition of 100 $s^{-1}$, using the double gap geometry. (a) Transient viscosity and (b) corresponding transient shear stress as a function of time for constant shear rates (0.1, 0.5, 1, 5, and 10 $s^{-1}$). The system exhibits pronounced shear-thinning behavior and time-dependent viscosity, particularly at lower shear rates, indicative of a complex fluid microstructure undergoing slow relaxation or structural evolution under shear. As discussed in the main text, this shear-thinning response arises from an additional torque read by the rheometer due to LF's interfacial adsorption at the air-liquid interface and cannot be avoided using the double-gap geometry.

Figure S12 – Steady-shear viscosity as a function of shear rate for LF (25 °C) or dLF at 65 °C, 80 °C or 95 °C), measured using the cone-and-plate (CP) geometry.

Figure S13 – Oscillatory interfacial time sweep (a) obtained using a bicone geometry (BiC68-5) positioned at the air-liquid interface of LF (25 °C) dispersion at 0.5 wt% and 25 °C, at a constant shear strain of 0.3% and frequency of 6.28 rad $s^{-1}$. From the beginning, a solid viscoelastic film is formed with $G' > G''$. Although the film does not reach an equilibrium condition after 140 min, the experiment was stopped to avoid sample evaporation effects. Oscillatory interfacial shear strain sweep (b) for LF (25 °C) at 0.5 wt% and 25 °C after

equilibration for 140 min, obtained at 6.28 rad $s^{-1}$. (c) Rotational steady-state interfacial viscosity for LF (25 °C) at 0.5 wt% and 25 °C after equilibration for 140 min.

Figure S14 – Steady-shear viscosity as a function of shear rate for (a) LF (native or heat-treated, at 65 °C, 80 °C or 95 °C) at 0.5 wt% in the presence of the surfactant Triton X-100 (TRX-100) at 0.001 wt%, measured using the double-gap (DG) geometry. Visual images of LF/BSM complexes (b) in the presence of the surfactant Triton X-100 (TRX-100) at 0.001 wt%, show how after some minutes, macroscopic phase separation is observed for all samples [*I.* LF (25 °C)/BSM/TRX-100, *II.* dLF (65 °C)/BSM/TRX-100, *III.* dLF (80 °C)/BSM/TRX-100, *IV.* LF (95 °C)/BSM/TRX-100]. Similar behavior was observed for the samples LF/BSM in the presence of sodium dodecyl sulfate (SDS), another surfactant (data not shown). Viscosity for the LF/BSM complexes shown in (b) was not measured as phase separation was observed due to interactions between the surfactant and mucin.

Table S1 - $\zeta$-potential values measured for 0.001 wt% samples of BSM, unheated lactoferrin (LF), and heated lactoferrin (dLF) at 65°C, 80°C and 95°C for 30 min. The reported values represent the mean value and standard deviation of three readings for two different samples (n = 3 × 2).

Table S2 – Boussinesq (*Bo*) number calculation for each geometry used in the present work. The information for each geometry is described in the materials and methods section.

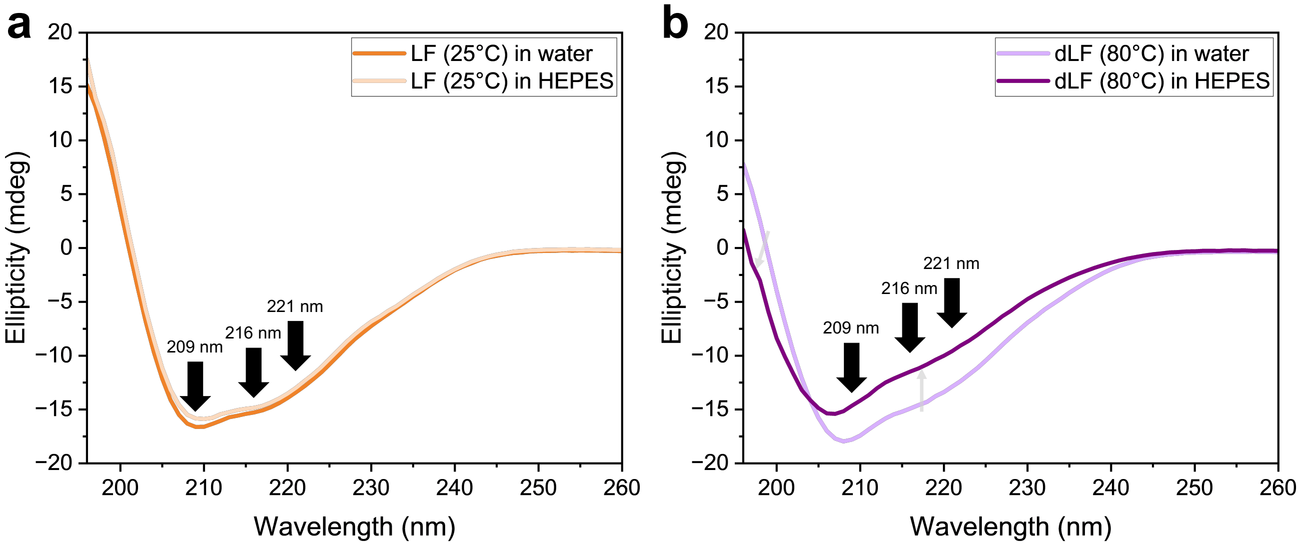


Figure S1 – Circular dichroism (CD) spectra for lactoferrin (LF) samples in water *versus* 10 mM HEPES buffer at pH 7.0 for (a) LF (25 °C) and (b) denatured lactoferrin (dLF) (80 °C), with black arrows indicating characteristic peak values for $\alpha$-helical and $\beta$-sheet structures and grey arrows indicating the shift caused by HEPES buffer. Data are reported as means of three replicates for each of two independent samples measured on separate days (n = 3 × 2).

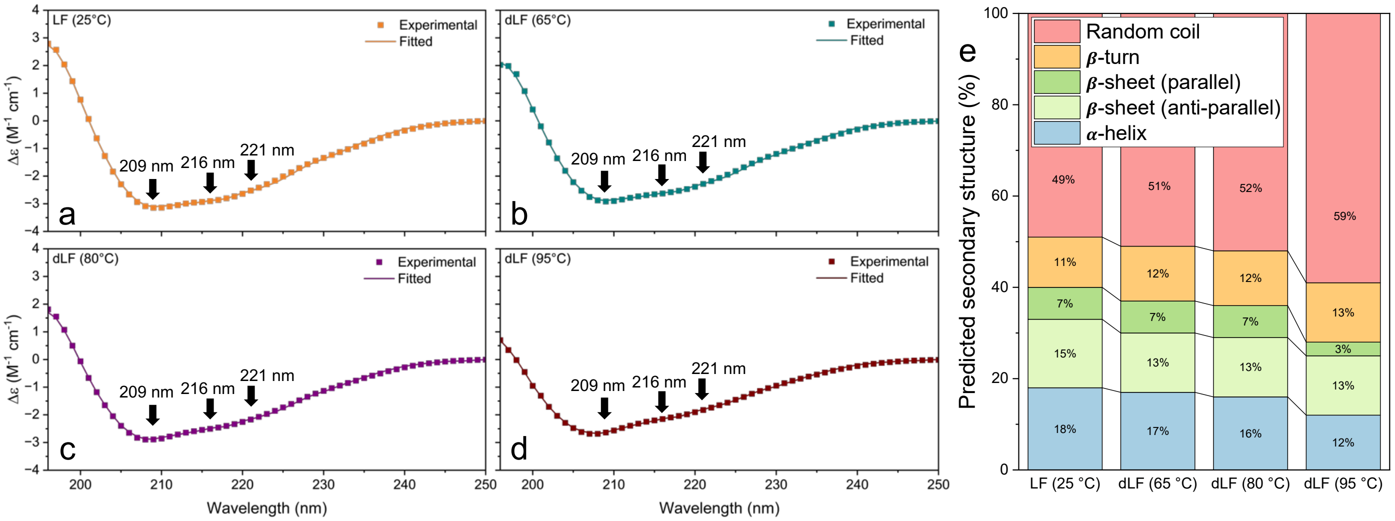


Figure S2 – CD experimental data and corresponding fits obtained using BeStSel to determine the secondary structure components of (a) LF (25 °C), (b) dLF (65 °C), (c) dLF (80 °C), (d) dLF (95 °C). The structural composition based on each fit for LF or dLF is represented in (e).

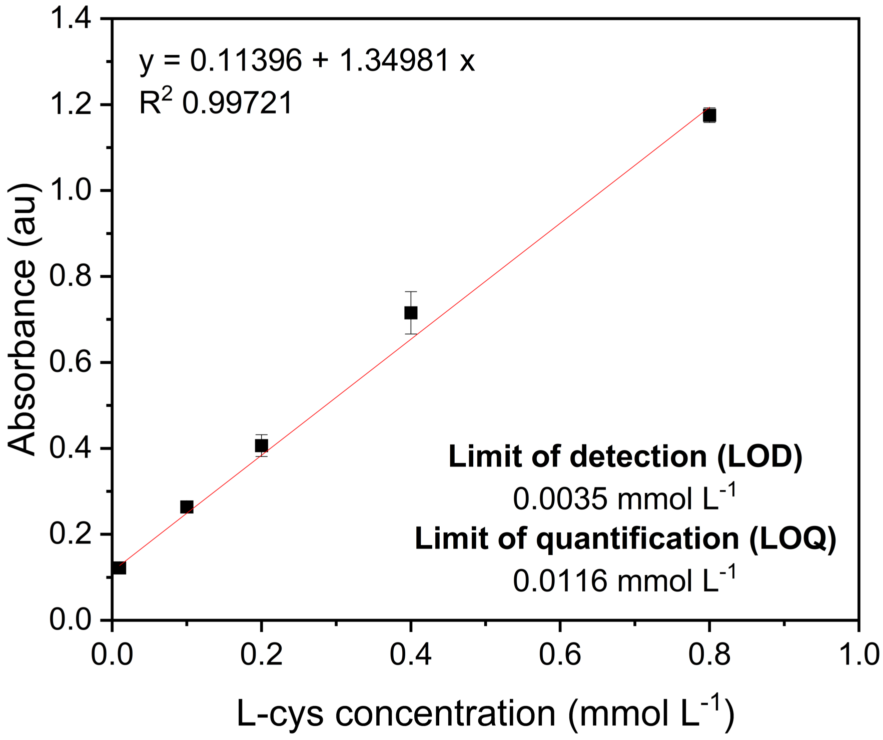


Figure S3– Calibration curve for thiol quantification using L-cysteine (L-cys) standards. The absorbance at 412 nm was plotted against L-cysteine concentration, yielding a linear fit with the equation $Absorbance = 0.11396 + 1.34981[Thiol\ concentration]$ and coefficient of determination $R^2$ of 0.99721. For LF and heat-treated LF at 65 °C, 80 °C or 95 °C for 30 min [dLF], no free thiol groups were detected as all absorbance values were below the limit of detection (LOD) of 0.0035 mmol $L^{-1}$. For BSM, an absorbance of 0.31 was read, corresponding to 0.15 mmol $L^{-1}$ of thiol groups. BSM has molecular weights ranging between $1 \times 10^7$ to $5 \times 10^7$ g/mol determined using AF4 (see Figure 3c), and considering a glycosylation contribution of 50-80% on the molecular weight yields 30 - 375 thiol groups per mucin molecule, which is in agreement with the 262 cysteine residues found in bovine MUC5B (UniProt ID F2FB42)[1]. Each sample/standard was read in triplicate for three different samples (n = 3 x 3) and results are reported as mean ± standard deviation.

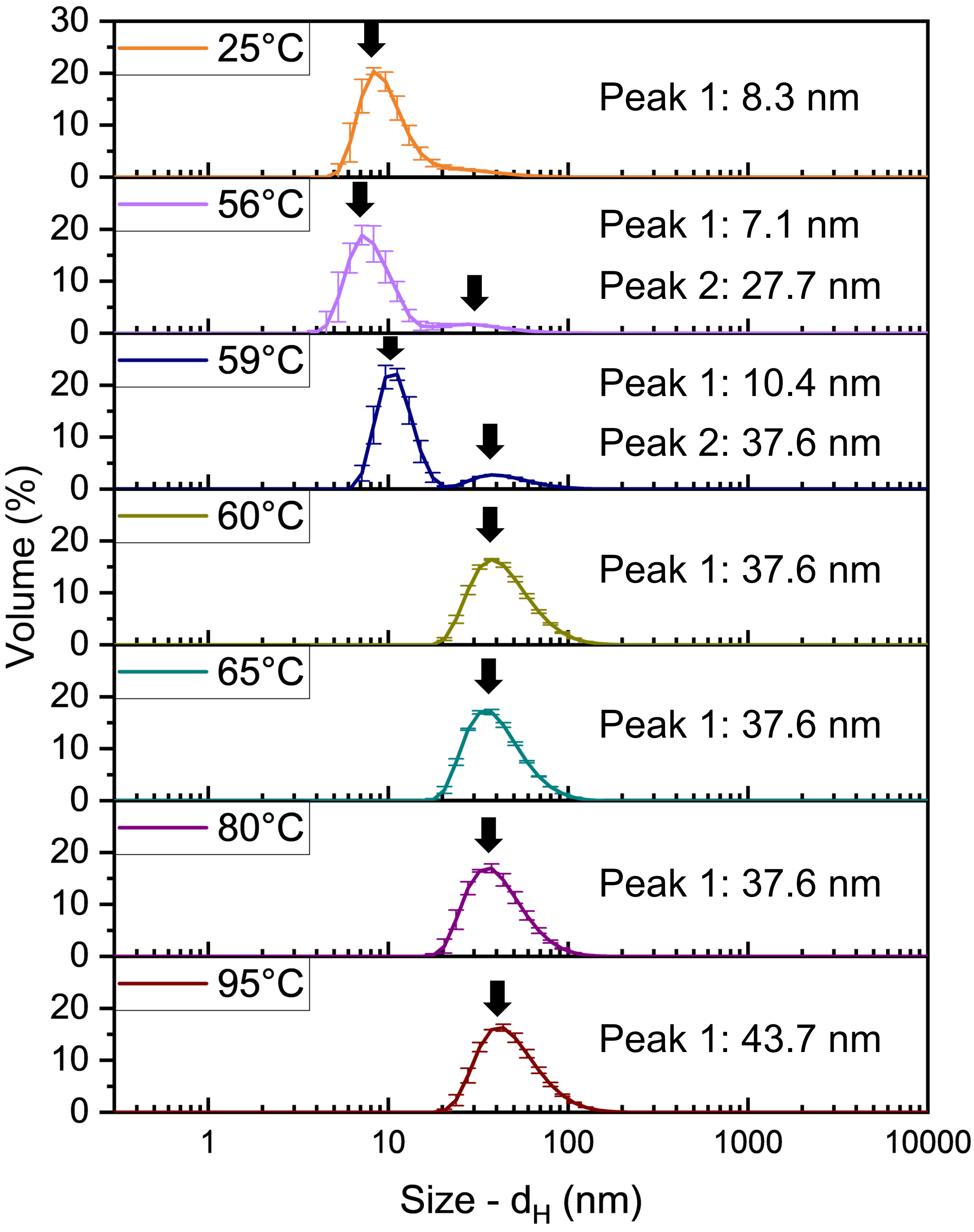


Figure S4 – Particle size distribution obtained by dynamic light scattering (DLS) at 25°C for 0.01 wt% LF in 10 mM HEPES buffer at pH 7.0 showing the sudden transition between 59 and 60 °C when all monomer sizes of LF disappear and are no longer observed after 60 °C, indicating protein denaturation and irreversible aggregation. Data represents means ± standard deviation of two replicates measured in triplicate ($n = 3 \times 2$).

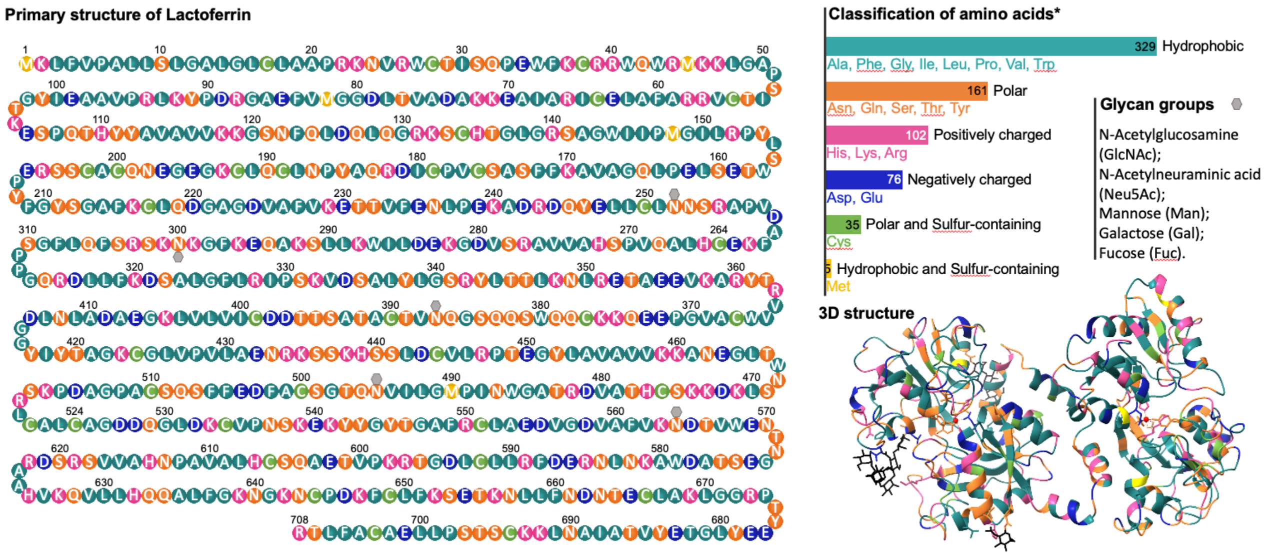


Figure S5 – Schematic representation of the primary structure of bovine (*Bos taurus*) lactoferrin, drawn based on the UniProt entry P24627[2]. The amino acid numbers for signal peptides are 1 to 19, of which one is a cysteine. Without the signal peptides, bovine lactoferrin contains 689 amino acids. In this representation, each small circle accounts for one amino acid, and these amino acids can be classified into different groups following the properties of their side chain. Here, we adopted this classification[3] to group the amino acids based on their polarity (hence yielding two classes – polar and nonpolar or hydrophobic amino acids) and charge (positively and negatively charged amino acids). The sulfur-containing amino acids are here separated in two different categories – "polar and Sulfur-containing" and "hydrophobic and Sulfur-containing", given the property of free thiols from cysteine groups to interact with mucin. The potential glycosylation sites are typically occupied by *N*-linked glycans and if the 19 signal-sequence amino acids are not shown, the glycosylated asparagine sites are 233, 281, 368, 478, and 547[4]. The one and three-letter symbols and names for the amino acids represented are: A – alanine – ala, C – cysteine – cys, D – aspartic acid – asp, E – glutamic acid – glu, F – phenylalanine – phe, G – glycine – gly, H – histidine – his, I – isoleucine – ile, K – lysine – lys, L – leucine – leu, M – methionine – met, N – asparagine – asn, P – proline – pro, Q – glutamine – gln, R – arginine – arg, S – serine – ser, T – threonine – thr, V – valine – val, W – tryptophan – trp, Y – tyrosine – tyr. The color-coded 3D representation based on each amino acid character was generated using ChimeraX[5].

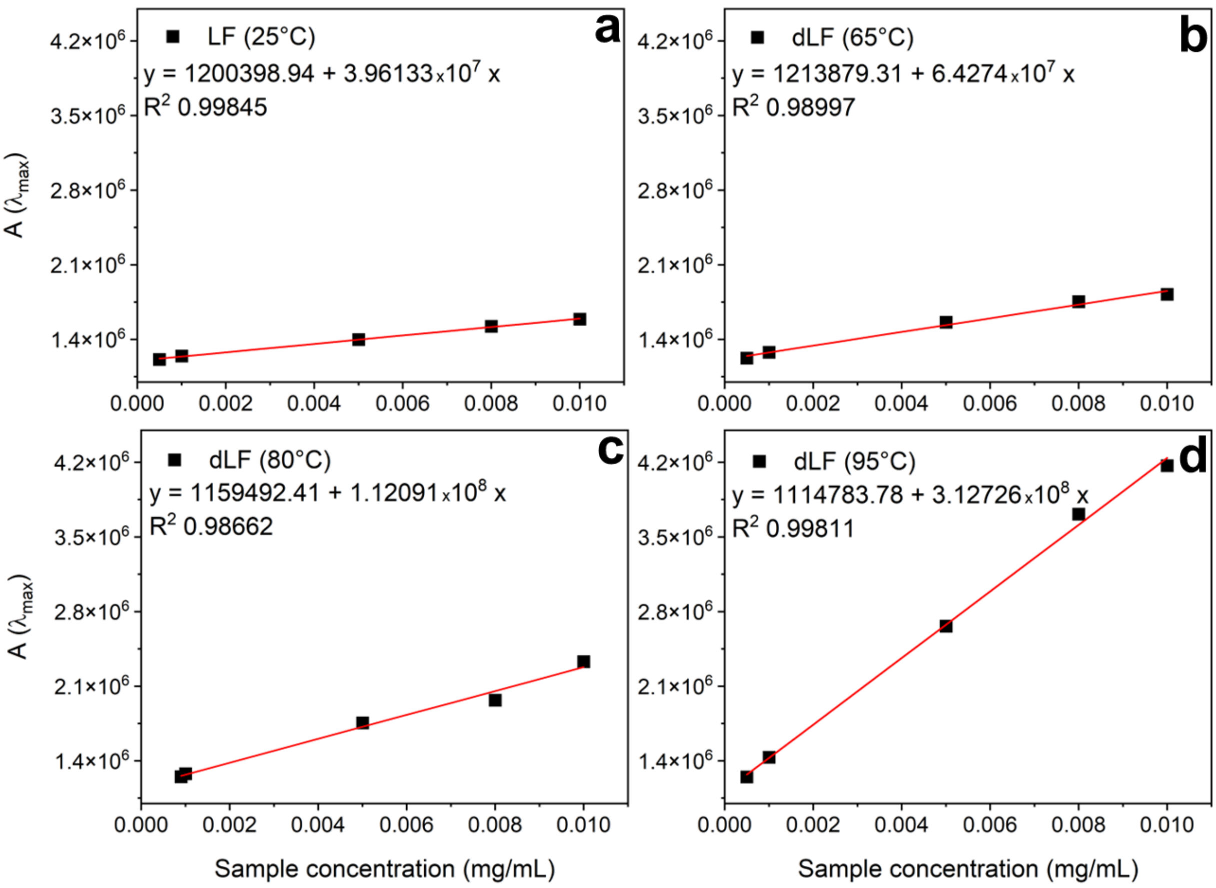


Figure S6 – Linear relationship observed between different concentrations of LF and their respective fluorescence emission measured at $\lambda$=491 nm after reacting unheated (a) or heat-treated LF at 65, 80 or 95°C (b), (c), and (d), respectively, with the fluorescent probe 8-Anilinonaphthalene-1-sulfonic acid (ANS). The angular coefficient of each linear regression corresponds to the surface hydrophobicity index ($H_0$) of each sample, as discussed in the main text. Measurements were performed in triplicate for three samples (n = 3 × 3) and results are reported as mean ± standard deviation for all nine readings.

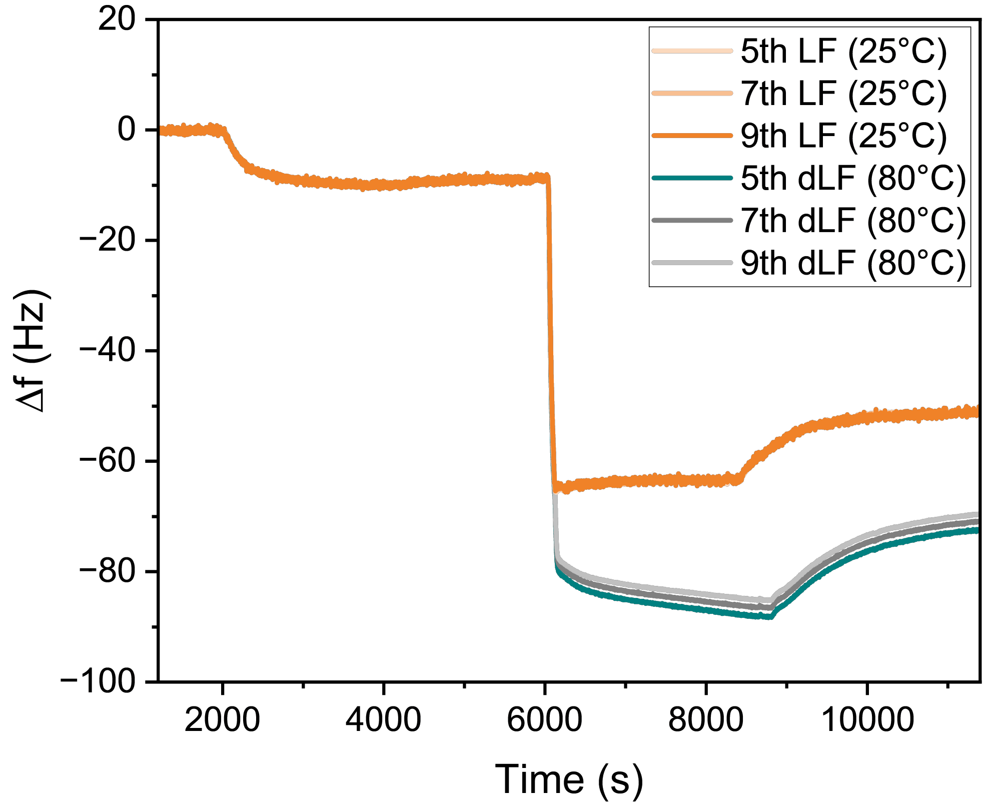


Figure S7 – Quartz crystal microbalance with dissipation monitoring (QCM-D) showing the frequency shift for the 5$^{th}$, 7$^{th}$ and 9$^{th}$ overtones as a function of time, illustrating the sequential adsorption of bovine submaxillary mucin (BSM) and filtered lactoferrin (LF) samples onto PDMS-coated $SiO_2$ sensors. A stable baseline at $\Delta f = 0$ is obtained for HEPES buffer. Then, a BSM dispersion is injected at ~2000s in the QCM-D chamber, which leads to a decrease in $\Delta f$ indicating mucin adsorption, followed by rinsing with HEPES buffer at ~4000s. Subsequently, filtered LF samples (by 0.22 $\mu$m hydrophilic membranes), either unheated LF (25 °C) or heat-treated, dLF (80 °C) for 30 min, were introduced. The last solution added was HEPES buffer at ~8280s.

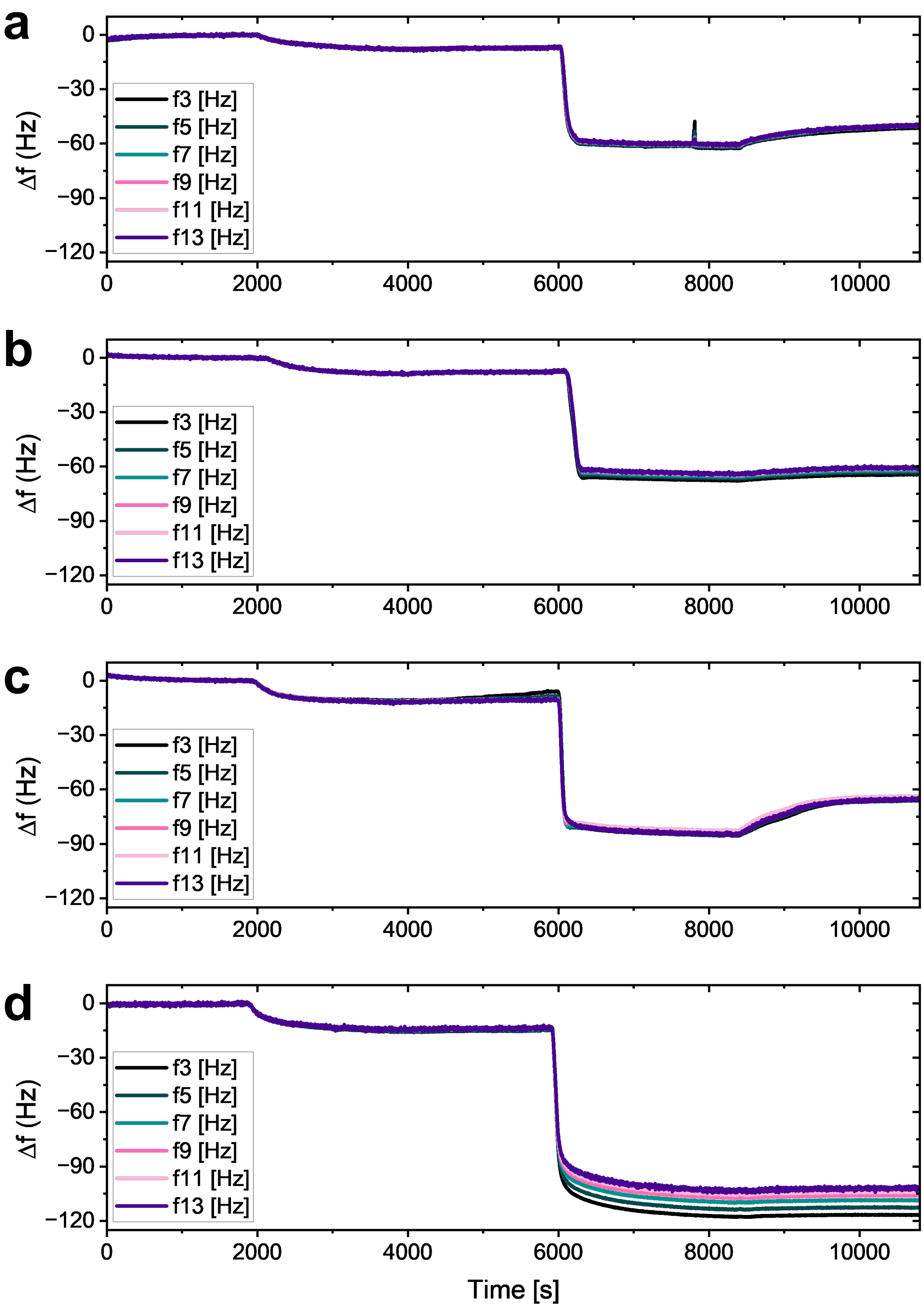


Figure S8 – QCM-D frequency shift showing raw data for one sensor each for (a) untreated LF (25 °C) or heat-treated (b) dLF (65 °C), (c) dLF (80 °C), or (d) dLF (95 °C) for 30 min, showing overtones from the 3rd (f3) to the 13th (f13) as a function of time. BSM dispersions were injected at ~2000s in the QCM-D chamber, followed by rinsing with HEPES buffer at ~4000s. Subsequently, LF or dLF samples were introduced at 6000s. The last solution added was HEPES buffer at ~8280s.

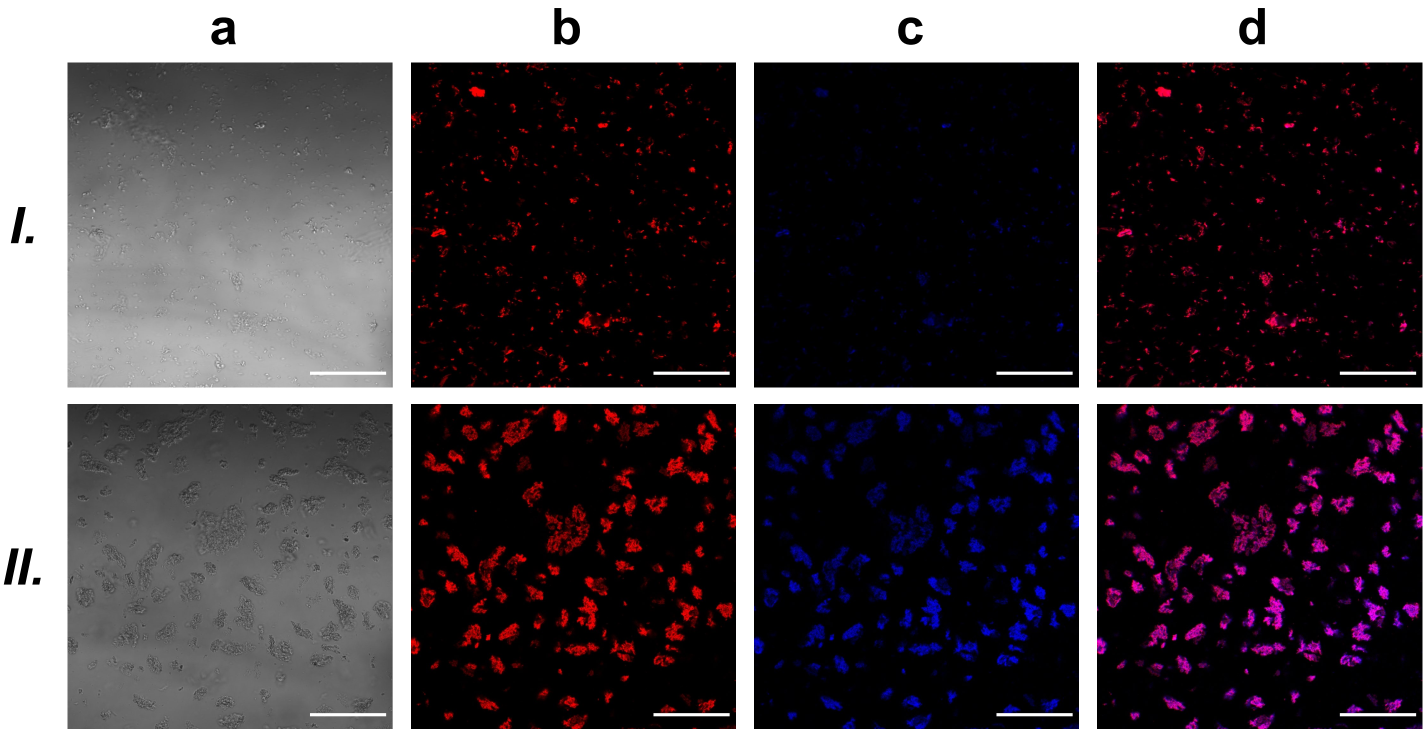


Figure S9 – Confocal micrographs of LF *I.* and BSM *II.* at 0.5 wt%. Each LF or BSM was prepared in the presence of 200 ppm of calcofluor white and 200 ppm of fast green, excited at 360 nm and 633 nm, respectively. (a) Transmitted light channel (b) fast green channel (proteinaceous structures) (c) calcofluor white channel (glycan structures) (d) composite of the fast green and calcofluor white channels. The white scale bar in each image represents 50 $\mu$m. Images were obtained with a 40x oil-immersed objective.

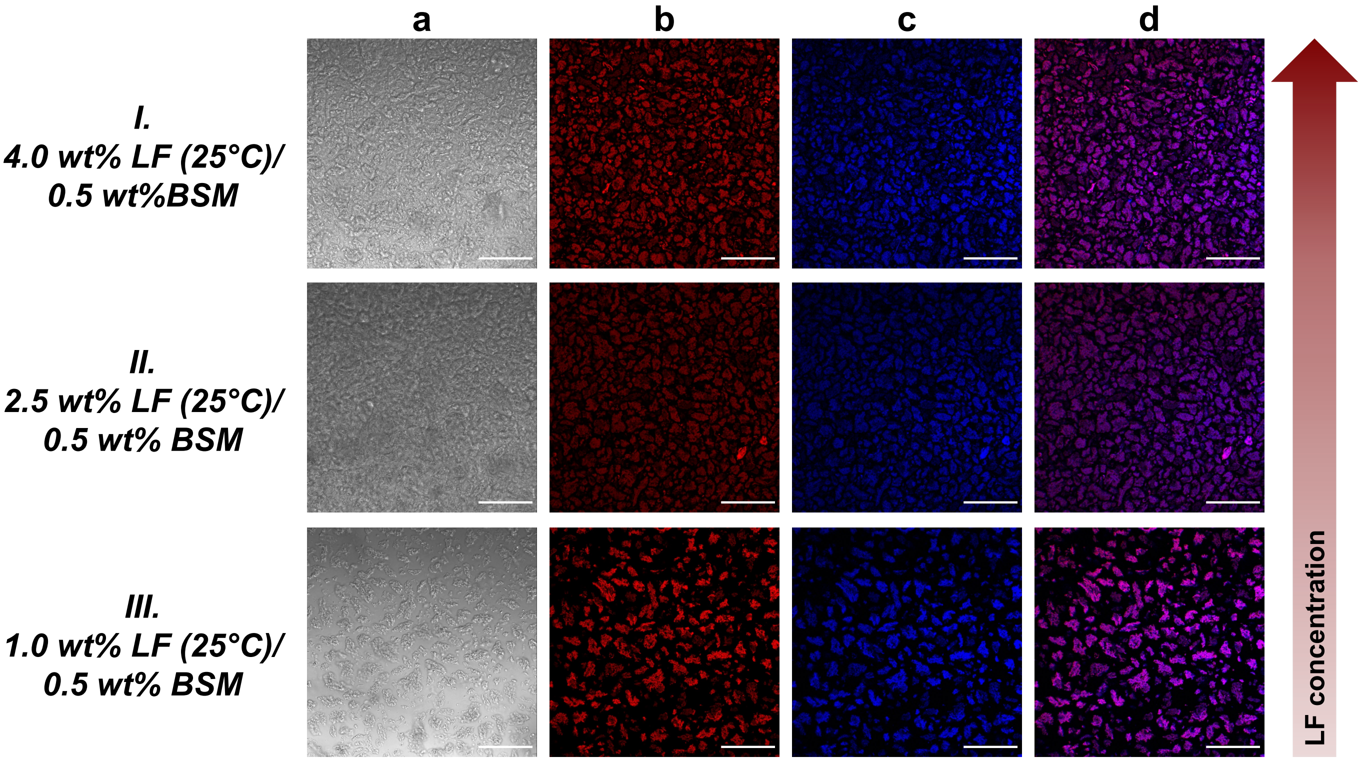


Figure S10 – Confocal micrographs of BSM/LF (25 °C) mixtures with 0.5 wt% BSM and *I.* LF at 4.0 wt% (highest concentration), *II.* LF at 2.5 wt%, and *III.* LF at 1.0 wt% (lowest concentration). Both LF and BSM were prepared in the presence of the staining agent - 200 ppm of fast green in the case of LF and 200 ppm of calcofluor white in the case of BSM, which were excited at 633 and 360 nm, respectively. The white scale bar in each image represents 50 $\mu$m. Images were obtained with a 40x oil-immersed objective, and the channels represented are (a) transmitted light, (b) fast green, (c) calcofluor white, (d) fast green and calcofluor white composite.

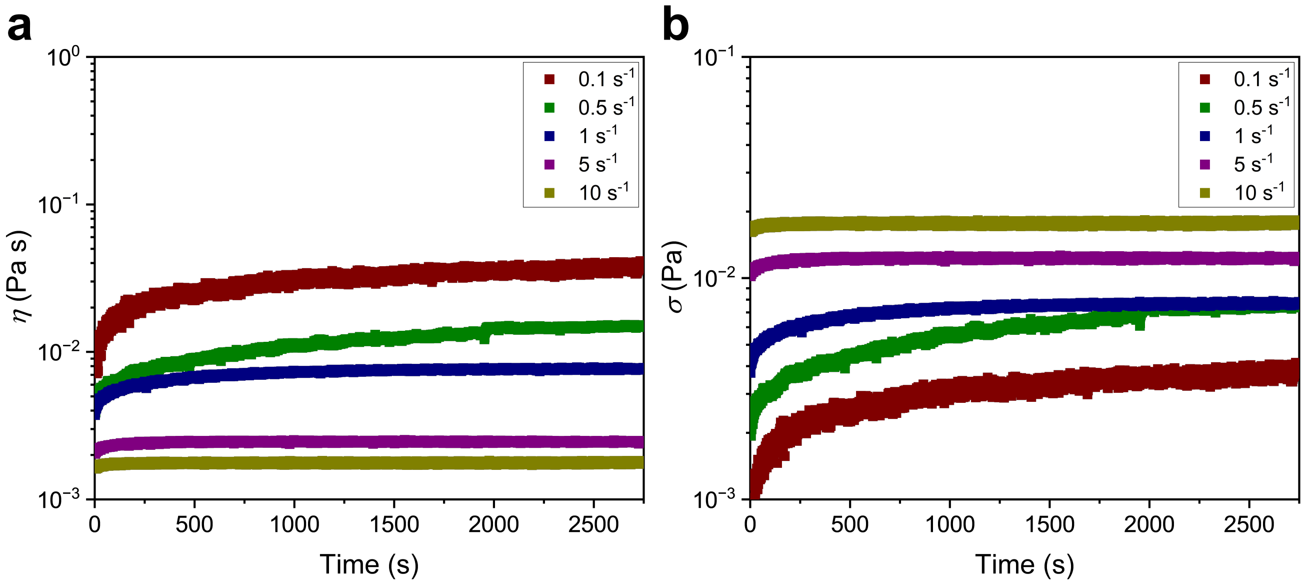


Figure S11 - Time-dependent rheological behavior of LF (25 °C) under steady shearing conditions, following a pre-shearing condition of 100 $s^{-1}$, using the double gap geometry. (a) Transient viscosity and (b) corresponding transient shear stress as a function of time for constant shear rates (0.1, 0.5, 1, 5, and 10 $s^{-1}$). The system exhibits pronounced shear-thinning behavior and time-dependent viscosity, particularly at lower shear rates, indicative of a complex fluid microstructure undergoing slow relaxation or structural evolution under shear. As discussed in the main text, this shear-thinning response arises from an additional torque read by the rheometer due to LF's interfacial adsorption at the air-liquid interface and cannot be avoided using the double-gap geometry.

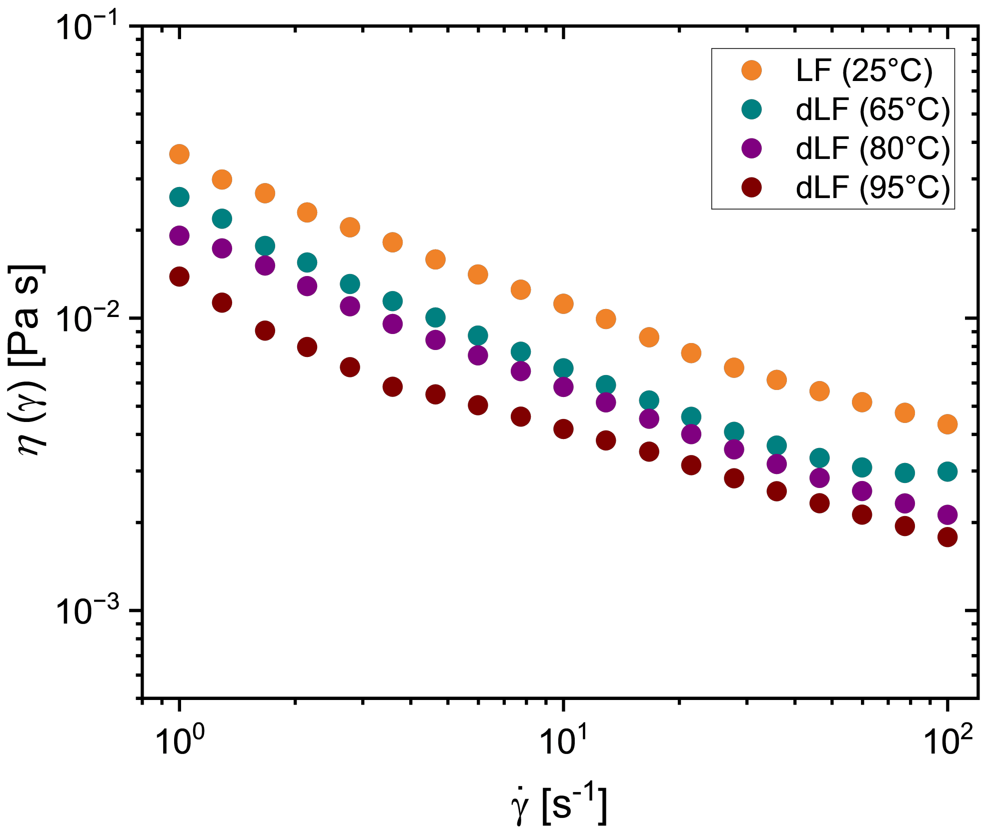


Figure S12 – Steady-shear viscosity as a function of shear rate for LF (25 °C) or dLF at 65 °C, 80 °C or 95 °C), measured using the cone-and-plate (CP) geometry.

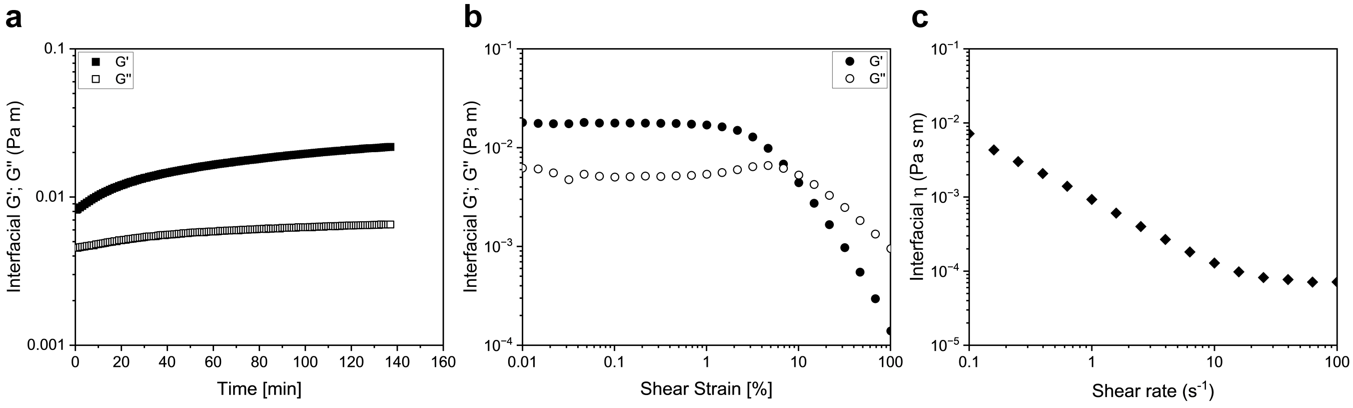


Figure S13 – Oscillatory interfacial time sweep (a) obtained using a bicone geometry (BiC68-5) positioned at the air-liquid interface of LF (25 °C) dispersion at 0.5 wt% and 25 °C, at a constant shear strain of 0.3% and frequency of 6.28 rad $s^{-1}$. From the beginning, a solid viscoelastic film is formed with G′ > G″. Although the film does not reach an equilibrium condition after 140 min, the experiment was stopped to avoid sample evaporation effects. Oscillatory interfacial shear strain sweep (b) for LF (25 °C) at 0.5 wt% and 25 °C after equilibration for 140 min, obtained at 6.28 rad $s^{-1}$. (c) Rotational steady-state interfacial viscosity for LF (25 °C) at 0.5 wt% and 25 °C after equilibration for 140 min.

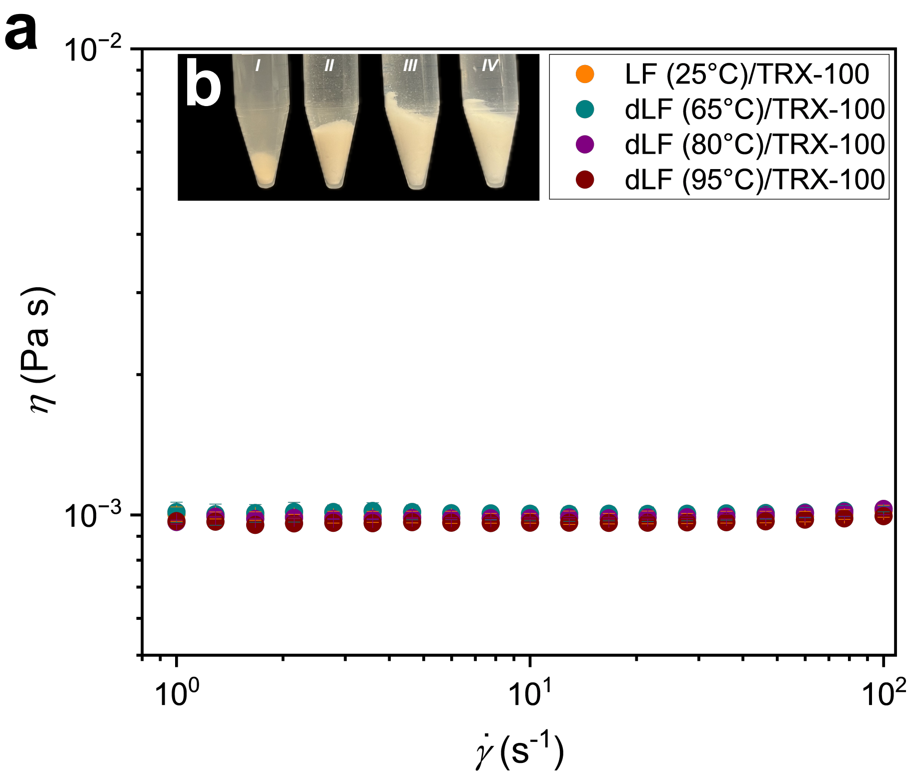


Figure S14 – Steady-shear viscosity as a function of shear rate for (a) LF (native or heat-treated, at 65 °C, 80 °C or 95 °C) at 0.5 wt% in the presence of the surfactant Triton X-100 (TRX-100) at 0.001 wt%, measured using the double-gap (DG) geometry. Visual images of LF/BSM complexes (b) in the presence of the surfactant Triton X-100 (TRX-100) at 0.001 wt%, show how after some minutes, macroscopic phase separation is observed for all samples [*I.* LF (25 °C)/BSM/TRX-100, *II.* dLF (65 °C)/BSM/TRX-100, *III.* dLF (80 °C)/BSM/TRX-100, *IV.* LF (95 °C)/BSM/TRX-100]. Similar behavior was observed for the samples LF/BSM in the presence of sodium dodecyl sulfate (SDS), another surfactant (data not shown). Viscosity for the LF/BSM complexes shown in (b) was not measured as phase separation was observed due to interactions between the surfactant and mucin.

Table S1 - $\zeta$-potential values measured for 0.001 wt% samples of BSM, unheated lactoferrin (LF), and heated lactoferrin (dLF) at 65°C, 80°C and 95°C for 30 min. The reported values represent the mean value and standard deviation of three readings for two different samples (n = 3 × 2).

| Sample | $\zeta$-potential in water, at pH 7.0 | $\zeta$-potential in HEPES, at pH 7.0 |
|---|---|---|
| BSM | -39.0 ± 0.65 mV | -25.5 ± 1.26 mV |
| LF (25 °C) | +20.5 ± 0.64 mV | +9.7 ± 0.35 mV |
| dLF (65 °C, 30 min) | +24.7 ± 1.26 mV | +17.3 ± 0.25 mV |
| dLF (80 °C, 30 min) | +24.1 ± 1.54 mV | +17.4 ± 0.41 mV |
| dLF (95 °C, 30 min) | +24.4 ± 2.12 mV | +17.2 ± 0.98 mV |

Table S2 – Boussinesq (*Bo*) number calculation for each geometry used in the present work. The information for each geometry is described in the materials and methods section.

| Shear rate | Geometry | *Bo* number |
|---|---|---|
| 1 $s^{-1}$ | Cone and Plate (CP) | 20 |
| | Double-gap (DG) | 6 |
| 100 $s^{-1}$ | Cone and Plate (CP) | 3 |
| | Double-gap (DG) | 0.8 |

## References


(1) *UniProt database - Mucin-5B - F2FB42*. https://www.uniprot.org/uniprotkb/F2FB42/entry (accessed 31/03/2025).

(2) *UniProt database - Lactotransferin - P24627*. https://www.uniprot.org/uniprotkb/P24627/entry (accessed 20/02/2024).

(3) Hughes, M. L.; Dougan, L. The physics of pulling polyproteins: a review of single molecule force spectroscopy using the AFM to study protein unfolding. *Rep Prog Phys* **2016**, *79* (7), 076601. DOI: 10.1088/0034-4885/79/7/076601 From NLM Medline.

(4) Goodman, R. E.; Schanbacher, F. L. Bovine lactoferrin mRNA: Sequence, analysis, and expression in the mammary gland. *Biochemical and Biophysical Research Communications* **1991**, *180* (1), 75–84. DOI: https://doi.org/10.1016/S0006-291X(05)81257-4.

(5) Meng, E. C.; Goddard, T. D.; Pettersen, E. F.; Couch, G. S.; Pearson, Z. J.; Morris, J. H.; Ferrin, T. E. UCSF ChimeraX: Tools for structure building and analysis. *Protein Sci* **2023**, *32* (11), e4792. DOI: 10.1002/pro.4792 From NLM Medline.
